# *In utero* exposure to the endocrine disruptor di-(2-ethylhexyl) phthalate promotes local adipose and systemic inflammation in adult male offspring

**DOI:** 10.1038/nutd.2014.13

**Published:** 2014-05-05

**Authors:** E Campioli, D B Martinez-Arguelles, V Papadopoulos

**Affiliations:** 1 Research Institute of the McGill University Health Centre, Montreal General Hospital, Montréal, QC, Canada; 2 Department of Medicine, McGill University, Montréal, QC, Canada; 3 Department of Biochemistry, McGill University, Montréal, QC, Canada; 4 Department of Pharmacology and Therapeutics, McGill University, Montréal, QC, Canada

## Abstract

**Background:**

Di-(2-ethylhexyl) phthalate (DEHP) is a plasticizer used to increase the flexibility of polyvinyl chloride. DEHP and its active metabolite mono-(2-ethylhexyl) phthalate are detected in many biological fluids during fetal and postnatal life. In rodent models, *in utero* DEHP exposure has been shown to alter sexual organ development, decrease testosterone and aldosterone production, increase body and epididymal adipose tissue weight, and raise serum lipids and glucose levels in male offspring.

**Objectives:**

The objective of this study is to characterize the effects of *in utero* DEHP exposure on adipose tissue development and function in male offspring.

**Methods:**

Sprague–Dawley pregnant dams were gavaged 1, 20, 50 or 300 mg DEHP per kg per day from gestational day 14 until birth.

**Results:**

Global gene expression analyses of postnatal day 60 male offspring that were exposed *in utero* to 300 mg DEHP per kg per day revealed increased expression of immune response and inflammation markers, and increased expression of differentiation pathway genes in the epididymal whole-adipose tissue and isolated stromal vascular fraction. C-reactive protein and tumor necrosis factor (TNF) serum levels were increased in the 300 mg DEHP *in utero*-exposed offspring. TNF levels in adipose tissue homogenates were increased in the 50 and 300 mg DEHP *in utero*-exposed offspring. Immunofluorescence studies revealed focal macrophage infiltration in whole-adipose tissue confirmed by increased CD163 tissue content.

**Conclusions:**

*In utero* DEHP exposure promotes local adipose tissue inflammation and chronic low-grade systemic inflammation. Moreover, evidence is presented, suggesting that DEHP increases the differentiation capacity of the pre-adipocytes of male offspring without affecting total body weight.

## Introduction

The use of phthalates as plasticizers began in 1933^1^ and production in recent years has reached five million tons,^2^ resulting in continuous and widespread exposure to the population.^2^ Phthalates are used in industry to increase polyvinyl chloride polymer flexibility, pliability and elasticity. They are found in industrial plastic, household items, paints, children's toys, personal care products and medical devices.^1^ Di-(2-ethylhexyl) phthalate (DEHP) is among the most-produced phthalates each year, with an estimated two million tons.^1^ As DEHP is not a part of the polyvinyl chloride polymer,^1, 2^ it is released into the environment^1^ and is absorbed mainly through dermal contact and ingestion.^3^ Parenteral exposure from medical devices and nutritional exposure account for the highest recorded exposures.^1, 2^ DEHP and its metabolites have endocrine-disrupting properties and are found in many biological fluids from fetuses and adults, raising human health concerns.^4, 5, 6, 7, 8^ Mono-(2-ethylhexyl) phthalate (MEHP) is the active metabolite of DEHP and has been shown to activate the peroxisome proliferator-activated receptor (PPAR) system, which is involved in lipid and glucose metabolism, adipocyte differentiation and development.^9, 10^

In men, there is an inverse correlation between levels of DEHP metabolites in urine and circulating androgen levels.^11^ *In utero* DEHP exposure exerts short- and long-term effects that are longer lasting when exposure occurs during fetal life.^10, 12^ *In utero* DEHP exposure alters sexual organ development in male rats and decreases fetal testosterone production.^13, 14^ We previously reported that *in utero* DEHP exposure reduced circulating testosterone and aldosterone levels in adult male offspring.^13, 15, 16^ Reduced testosterone levels (primary hypogonadism) have been linked to obesity,^17^ osteoporosis^18^ and cardiovascular diseases.^19^ The effect of phthalates on pre-adipocyte differentiation is well documented *in vitro* and *in vivo*. We previously reported that MEHP increases differentiation of the human pre-adipocyte SW872 cell line.^20^ MEHP was also shown to activate PPAR-γ and promote adipogenesis in murine 3T3-L1 cells^21^ and human primary pre-adipocytes.^22^ Similar effects were found *in vivo* when perinatal exposure to DEHP or MEHP increased body and epididymal adipose tissue weight, serum lipids and glucose levels in postnatal day (PND) 60 offspring.^23, 24^ A recent study demonstrated that intraperitoneal injection of DEHP increased levels of the differentiation markers *Pparg*, *Ap2* and *Fas* in 6-week-old C57BL/6J male mice.^24^

Based on these observations, we hypothesized that *in utero* DEHP exposure affects the development and function of the adipose tissue. We report herein that *in utero* DEHP exposure increases the differentiation capacity of pre-adipocytes and promotes local adipose tissue inflammation and chronic low-grade systemic inflammation.

## Materials and methods

### Animals and treatment

Timed pregnant Sprague–Dawley rats were purchased from Charles River Laboratories (Saint-Constant, QC, Canada) and gavaged daily with corn oil or 1, 20, 50 or 300 mg DEHP per kg per day (Sigma-Aldrich Canada Ltd, Oakville, ON, Canada) from gestational day 14 until parturition (corresponding to PND0). Pregnant dams were weighed every 2 days and doses were adjusted accordingly. Male offspring were killed at PND60 and epididymal adipose tissue was snap-frozen in liquid nitrogen or fixed in 4% paraformaldehyde (MJS BioLynx Inc., Brockville, ON, Canada). Animals were handled according to protocols approved by the McGill University Animal Care and Use Committee. The doses of DEHP in our rat studies were calculated to correspond to human equivalent doses according to the formula proposed by Reagan-Shaw *et al.*^25^ Briefly, the dose translation from HED to mg kg^−1^ per day exposure in the rat is not based on a simple conversion from human to rodent body weight, but it relies on the concept of the body surface area. The body surface area factor is a unitless number, equivalent to the no-observed-adverse-effect-level on an mg m^−2^ basis.^26^ The use of the body surface area factor is preferable because it correlates better several biological parameters across mammalian species.^25^ Thus, humans are exposed to DEHP doses corresponding to 0.18–62.5 mg kg^−1^ per day rat exposures. Rodent data indicate a no-observed-adverse-effect-level for DEHP of 5 mg kg^−1^ per day, and lowest-observed-adverse-effect-level for developmental effects of 10 mg kg^−1^ per day.^2^ We chose DEHP doses (1–300 mg kg^−1^ per day) that include environmentally relevant doses for humans.

### Stromal vascular fraction isolation

Epididymal adipose tissue was washed with 1 × phosphate buffer saline (Life Technologies Inc., Burlington, ON, Canada) and visible blood vessels were removed from the fat pads. The minced tissues were digested for 1 h at 37 °C with type II collagenase solution (1.2 mg ml^−1^; Sigma-Aldrich Canada Ltd) and 2% bovine serum albumin fraction V (Roche Applied Science, Laval, QC, Canada) in phosphate buffer saline (pH 7.4) in an orbital shaking oven. Cell suspensions were centrifuged in polyethylene centrifuge tubes (Dow Corning Corporation, Midland, MI, USA) for 5 min at 250 × *g* and the floating layer composed of adipocytes was discarded. The pellet was resuspended in 5 ml erythrocyte lysis buffer (8.26 g NH_4_Cl, 1 g KHCO_3_ and 0.037 g EDTA in 1 liter water, pH 7.3; Sigma-Aldrich Canada Ltd) at room temperature for 5 min, followed by the addition of phosphate buffer saline (pH 7.4) to stop the lysis reaction. Cells were initially filtered through 100-μm nylon strainer and, subsequently, a 40-μm nylon strainer (BD Canada, Mississauga, ON, Canada). Finally, cell suspensions were centrifuged at 350 × *g* for 5 min at 4 °C and the pellet was stored at −80 °C until RNA extraction.

### RNA extraction and global gene expression analyses

Total RNA from the adipose tissue, stromal vascular fraction (SVF) and the liver were extracted using RNeasy Lipid Tissue Mini Kit (Qiagen Inc., Toronto, ON, Canada) according to the manufacturer's instructions. RNA concentration and purity were determined using the NanoDrop ND-1000 spectrophotometer (Thermo Fisher Scientific Inc., Mississauga, ON, Canada). Adipose tissue and SVF mRNAs were fluorescently tagged, reverse transcribed and hybridized to a Rat Genome 230 2.0 Array (Affymetrix, Santa Clara, CA, USA) at the McGill University and Genome Quebec Innovation Centre facilities. Data were normalized using the PLIER algorithm and the consecutive sampling method was used to ascertain differential expression. Three microarrays were hybridized for PND60 males exposed *in utero* to corn oil (vehicle) and 300 mg DEHP per kg per day, each from one pregnant dam. Pathway analyses and gene clustering of significantly changed genes were performed using the DAVID bioinformatics database.

### mRNA quantification using quantitative reverse transcription-PCR

cDNA synthesis and quantitative reverse transcription-PCR were performed as previously described.^20^ Table 1 shows the sequences and annealing temperatures for each primer pair. β-actin mRNA (*Actb*) was used as an internal reference gene.

### Serum enzyme-linked immunosorbent assay

Blood was collected by cardiac puncture. Serum was separated and enzyme-linked immunosorbent assays were used to quantify adiponectin (R&D Systems Inc., Minneapolis, MN, USA), CD163 (Cusabio Biotech, Cedarlane Laboratories Ltd, Burlington, ON, Canada), C-reactive protein (CRP; EMD Millipore Corporation, Billerica, MA, Canada), interleukin 6 (IL-6; R&D Systems Inc.), leptin (Life Technologies Inc.), plasminogen activator inhibitor-1 (Oxford Biomedical Research, Cedarlane Laboratories Ltd), resistin (Bertin Pharma, Cayman Chemical Company, Ann Arbor, MI, USA) and tumor necrosis factor (TNF; R&D Systems Inc.) according to the manufacturer's instructions. Absorbance was read at 450 nm in a VICTOR X5 Multilabel Plate Reader (PerkinElmer Inc., Waltham, MA, USA).

### Adipose tissue protein extraction

Proteins were extracted according to previously reported method.^27^ In brief, 100 mg adipose tissue was homogenized with 1 ml extraction buffer containing 50 mM MgCl_2_ (Sigma-Aldrich Canada Ltd), 0.05% bovine serum albumin (Roche Applied Science), 3% fetal bovine serum (Life Technologies Inc.) and 25 mM PIPES sodium salt (Sigma-Aldrich Canada Ltd) in phosphate buffer saline. The homogenate was centrifuged at 1000 × *g* for 10 min at 4 °C and the supernatant was collected and assayed for CD163, CRP, IL-6 and TNF as described above.

### Immunohistochemistry

Immunohistochemistry was performed in 5-μm paraffin sections of epididymal adipose tissue fixed in 4% paraformaldehyde as previously described.^15^ Tissue slides were incubated overnight in primary mouse IgG1 anti-CD163 monoclonal antibody solution (1:100 dilution; Acris Antibodies Inc., San Diego, CA, USA). Alexa Fluor 488 goat anti-mouse IgG was used as secondary antibody (1:300 dilution; Life Technologies Inc.). DAPI (4',6-diamidino-2-phenylindole) was used for nuclear staining. Negative control slides were prepared by incubating with the corresponding secondary antibody. Three pieces from different locations of the fat pad were analyzed for each sample (*n*=3).

### Glucose and C-peptide

Blood was collected by cardiac puncture after a 12-h overnight fast. Glucose levels were immediately measured using Bayer's CONTOUR NEXT meter (Bayer Inc., Toronto, ON, Canada). Serum was used to quantify by enzyme-linked immunosorbent assay the levels of C-peptide (Mercordia Inc., Uppsala, Sweden).

### Statistical analyses

Data are expressed as the mean±s.e.m. and were analyzed using one-way analysis of variance followed by Dunnett's *post hoc* tests or t-tests using the GraphPad Prism program (GraphPad Software Inc., La Jolla, CA, USA). *P*<0.05 (*, #), *P*<0.01 (**), and *P*<0.001 (***) were used as indicators of the level of significance. For all experiments, the experimental unit was the pregnant dam and in the event that more siblings were available, the individual values were averaged to obtain a unique value per dam.

## Results

### Whole-adipose tissue and SVF gene expression

**Figure 1:**
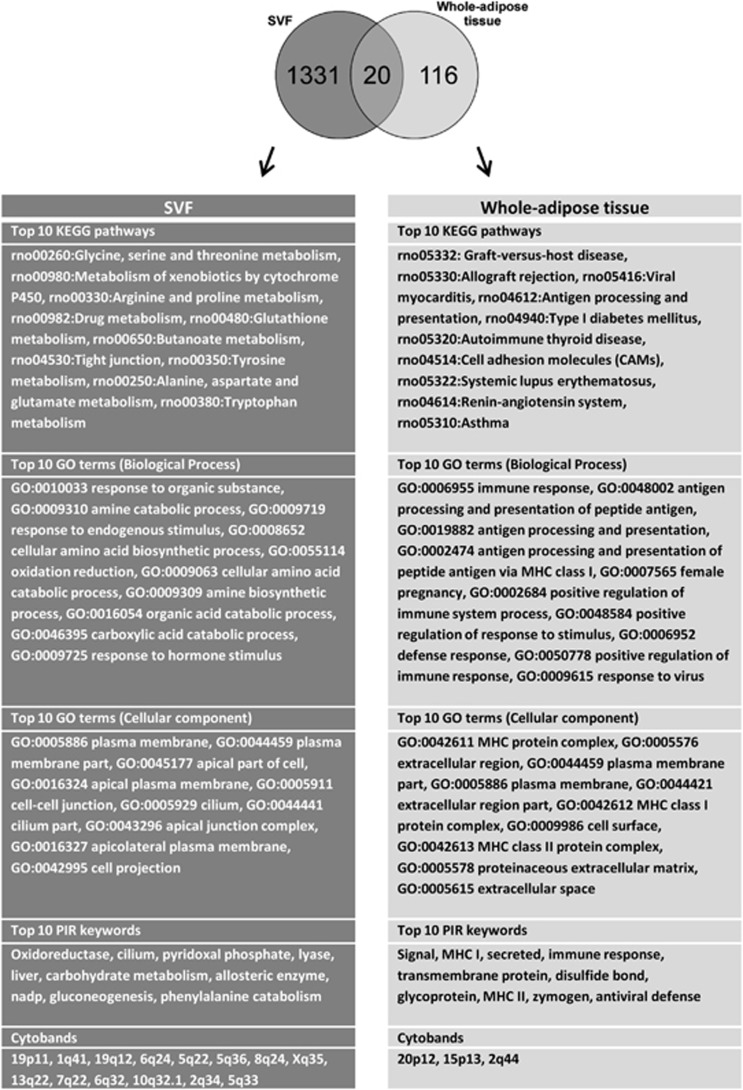
Global gene expression changes of the SVF and whole-adipose tissue in DEHP-exposed offspring. The Venn diagram shows the genes significantly changed by DEHP in the SVP and whole-adipose tissue. Tables show the top 10 pathways, GO terms, keywords and cytobands affected by *in utero* DEHP exposure.

To gain insight into the long-term effects of *in utero* DEHP exposure in whole-adipose tissue and SVF, we performed global gene expression studies. Epididymal whole-adipose tissue from male offspring exposed *in utero* to control or 300 mg DEHP per kg per day was collected at PND60. We observed 65 up- and 71 downregulated genes in whole-adipose tissue, whereas SVF had 181 up- and 1265 downregulated genes (Figure 1, Supplementary Tables S1 and S2). We analyzed the top 10 pathways, GO-term classifications and keywords associated with the significantly changing genes from whole-adipose and SVF samples, using the bioinformatics website DAVID. In whole-adipose tissue, DEHP primarily targeted the antigen-processing and -presenting pathway, characterized by upregulation of major histocompatibility complex class I and II molecules located in the chr20p12 locus (*RT1-EC2*, *RT1-S3*, *RT1-CE5*, *RT1-A1*, *RT1-Db1*, *RT1-EC2* and *RT1-Bb*). The effects of DEHP on the SVF were more pronounced and were characterized by a sharp downregulation of genes associated with metabolic pathways. DEHP affected the PPAR pathway in the SVF (*Olr1* and *Aqp7* were upregulated, whereas *Apoc3*, *Fads2*, *Gk*, *Acsl1*, *Slc27a2*, *Ehhadh* and *Scd* were downregulated). The renin angiotensin (AT) system (RAS) was also targeted by DEHP and characterized by downregulation of *Ang*, *Ctsg*, *Agtr2* and *Agtr1* in the SVF and upregulation of *Ctsg*, *Cma1* and *Cpa3* in whole-adipose tissue.

### Inflammatory markers gene expression

**Figure 2:**
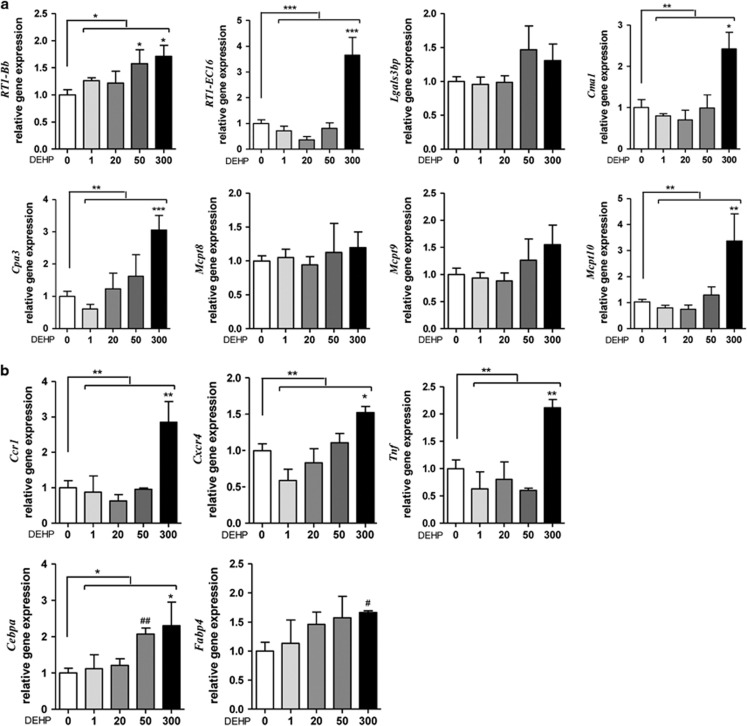
Effect of *in utero* DEHP exposure on gene expression in male offspring. (**a**) Total adipose tissue quantitative reverse transcription-PCR (qRT-PCR) products of *RT1-Bb*, *RT1-EC16*, *Lgals3bp*, *Cma1*, *Cpa3*, *Mcpt8*, *Mcpt9* and *Mcpt10* normalized to *Actb.* Data are presented as fold over control (*n*=7). (**b**) SVF qRT-PCR products of *Ccr1*, *Cxcr4*, *Tnf*, *Cebpa* and *Fabp4* normalized to *Actb.* Data are presented as fold over control (*n*=3). Results are expressed as the mean±s.e.m. One-way analysis of variance (ANOVA) followed by Dunnett's *post hoc* tests (*) or t-tests (^#^) was used to calculate statistical significance compared with control; ^#,^*P<0.05, ^##,^**P<0.01, ***P<0.001. Bars indicate doses significant by ANOVA; *P<0.05.

We confirmed the gene expression changes observed in the global gene expression results from PND60 male offspring whole-adipose tissue and SVF using quantitative reverse transcription-PCR . Exposure to 300 mg DEHP per kg per day induced *RT1-Bb* (1.7-fold, *P*<0.05), *RT1-E16* (17.4-fold, *P*<0.001), *Cma1* (2.4-fold, *P*<0.05), *Cpa3* (3.1-fold, *P*<0.001) and *Mcpt10* (3.4-fold, *P*<0.01) in whole-adipose tissue (Figure 2a). *RT1-Bb* showed a 1.6-fold increase (*P*<0.01) when animals were exposed to 50 mg DEHP per kg per day (Figure 2a). Gene expression from the SVF showed that animals exposed to 300 mg DEHP per kg per day induced *Ccr1* (2.9-fold, *P*<0.01), *Cxcr4* (1.5-fold, *P*<0.05), *Tnf* (2.1-fold, *P*<0.01), *Cebpa* (2.3-fold, *P*<0.05) and *Fabp4* mRNA levels (1.7-fold, *P*<0.05, *t*-test; Figure 2b). At 50 mg DEHP per kg per day, only *Cebpa* expression was increased by 2.1-fold (*P*<0.05, *t*-test; Figure 2b). There were no changes observed in *Crp*, *Il6* and *Tnf* mRNA levels in whole-adipose tissue (Figure 4a) or *Crp* expression in the liver (Figure 4b) from male offspring exposed to 300 mg DEHP per kg per day.

### Serum adipokine levels

**Figure 3:**
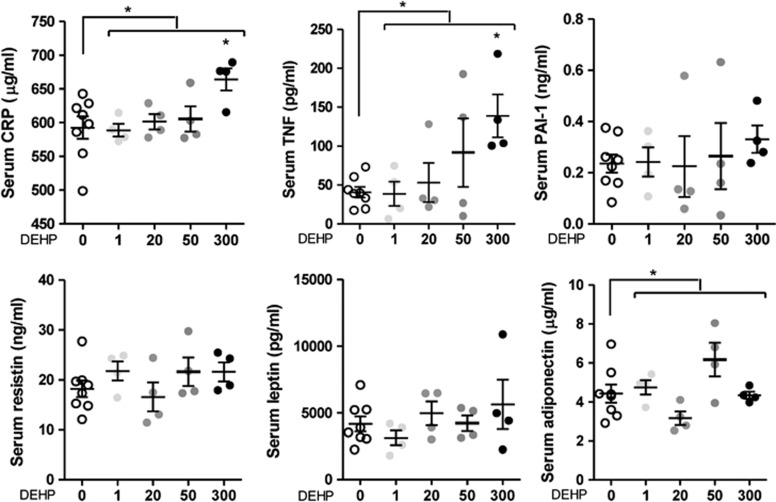
Effect of *in utero* DEHP exposure on male offspring serum adipokine concentration. Serum levels of CRP, TNF, plasminogen activator inhibitor (PAI-1), resistin, leptin and adiponectin are shown. Results are expressed as the mean±s.e.m. (*n*=4 except for control group where *n*=8). One-way analysis of variance (ANOVA) followed by Dunnett's *post hoc* tests was used to calculate statistical significance compared with control; *P<0.05. Bars indicate doses significant by ANOVA; *P<0.05.

To determine whether exposure to DEHP induced systemic inflammation or altered adipocyte endocrine function, we quantified circulating levels of inflammatory cytokines and adipokines. Male offspring exposed *in utero* to 300 mg DEHP per kg per day showed increased serum levels of CRP (664.20±16.54 μg ml^−1^, *n*=4, *P*<0.05) compared with controls (592.60±16.53 μg ml^−1^, *n*=8). TNF was also increased in the 300 mg DEHP per kg per day exposed offspring (139.00±27.56 pg ml^−1^, *n*=4, *P*<0.05) compared with controls (40.59±6.70 pg ml^−1^, *n*=8; Figure 3). Serum adiponectin levels followed a polynomial cubic trend (*F*-test, *P*=0.0105) and DEHP-induced changes were significant by analysis of variance (*P*<0.05); nonetheless, no specific DEHP dose reached the significance using Dunnett's *post hoc* test. IL-6 was undetectable in the samples; leptin, plasminogen activator inhibitor-1 and resistin were not affected by DEHP.

### Epididymal adipose tissue adipokine production

**Figure 4:**
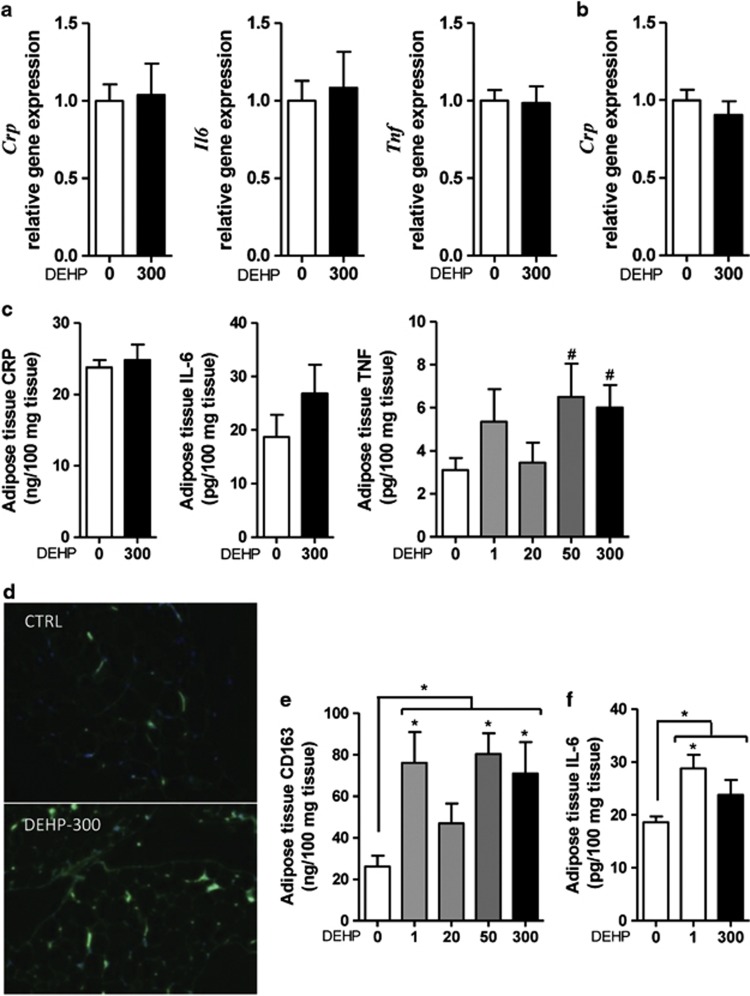
Effect of *in utero* DEHP exposure on male offspring hepatic and adipose tissue inflammatory marker expression and adipose tissue macrophage infiltration. (**a**) Total adipose tissue quantitative reverse transcription-PCR (qRT-PCR) products of *Crp*, *Il6* and *Tnf* normalized to *Actb.* Data are presented as fold over control (*n*=7). (**b**) Liver qRT-PCR products of *Crp* normalized to *Actb* and presented as fold over control (*n*=7). (**c**) Epididymal adipose tissue content of CRP, IL-6 and TNF as determined using enzyme-linked immunosorbent assay (ELISA; *n*=7, except the 50 mg DEHP group where *n*=5). (**d**) Representative histological sections of adipose tissue controls and treated with 300 mg DEHP per kg per day stained for CD163 (green) and DAPI (blue). Objective × 20. (**e**) Adipose tissue content of CD163 as determined by ELISA (*n*=5 except 300 mg DEHP group where *n*=6). (**f**) Retroperitoneal adipose tissue content of IL-6 as determined by ELISA (*n*=8, except the 1 mg DEHP group where *n*=4). Results are expressed as the mean±s.e.m., one-way analysis of variance (ANOVA) followed by Dunnett's *post hoc* tests (*) or *t*-tests (^#^) were used to calculate statistical significance compared with control; ^#,^ *P<0.05. Bars indicate doses significant by ANOVA; *P<0.05.

To evaluate whether whole-adipose tissue was a source for the increased serum cytokines, we quantified whole-adipose CRP, IL-6 and TNF levels. Offspring exposed to 50 or 300 mg DEHP per kg per day showed a significant increase in local levels of TNF (6.50±1.55 pg per 100 mg tissue, *n*=5, and 6.01±1.05 pg per 100 mg tissue, *n*=7, *P*<0.05, respectively) compared with the control group (3.10±0.57 pg per 100 mg tissue, *n*=7; Figure 4c). Local CRP and IL-6 levels did not significantly change. Similarly, mRNA expression of *Crp*, *Il6* and *Tnf* were not affected in whole-adipose tissue.

### Adipose tissue macrophage infiltration/activation

To evaluate the presence of macrophage infiltration and/or activation in whole-adipose tissue, we analyzed the monocyte/macrophage marker CD163 by immunofluorescence. CD163 along with mannose and integrin αvβ5 receptors were found in human adipose tissue macrophages and identified as macrophage type 2-like cells with excessive pro-inflammatory mediator production.^28^

There was macrophage infiltration in animals exposed to 300 mg DEHP per kg per day compared with controls (Figure 4d). The macrophage infiltration was not homogenous, but rather was in foci within the adipose tissue. To confirm the focal infiltration, adipose tissue lysates were quantified for CD163 levels. Offspring exposed to 1, 50 or 300 mg DEHP per kg per day showed a significant increase in CD163 levels (76.15±14.78 ng per 100 mg tissue, *n*=5; 80.41±9.94 ng per 100 mg tissue, *n*=5; and 70.94±15.20 ng per 100 mg tissue, *n*=6, *P*<0.05, respectively) compared with the control group (26.15±5.24 ng per 100 mg tissue, *n*=5; Figure 4e).

### Retroperitoneal adipose tissue adipokine production

To evaluate whether the inflammation pathway was activated in a different type of adipose tissue, we quantified the retroperitoneal expression levels of IL-6. Offspring exposed to 1 mg DEHP per kg per day showed a significant increase in local levels of IL-6 (28.79±2.58 pg per 100 mg tissue, *n*=4, *P*<0.05) compared with the control group (18.61±1.11 pg per 100 mg tissue, *n*=8; Figure 4f). The increased inflammatory response was then confirmed by measuring TNF levels by enzyme-linked immunosorbent assay in the 1 mg DEHP per kg per day group (12.61±2.28 pg per 100 mg tissue, *n*=4, *P*<0.05) compared with the control group (9.749±1.39 pg per 100 mg tissue, *n*=6).

### Metabolic profile

**Figure 5:**
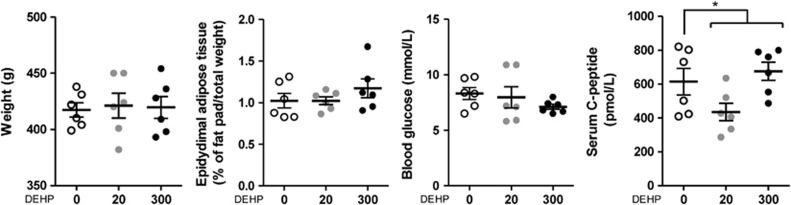
Effect of *in utero* DEHP exposure on male offspring total weight, epididymal adipose tissue pad weight, blood glucose and serum C-peptide. Results are expressed as the mean±s.e.m., *N*=6; one-way analysis of variance (ANOVA) followed by Dunnett's *post hoc* tests. Bars indicate doses significant by ANOVA; *P<0.05.

To evaluate the metabolic effects of *in utero* exposure to DEHP, we measured total and epididimal adipose tissue weight, glucose and insulin levels (C-peptide). There were no significant changes in total body or epididymal adipose tissue weight (Figure 5). C-peptide blood levels followed a polynomial quadratic trend (F-test, *P*= 0.0400) and the doses were significant by analysis of variance (*P*<0.05); however, no specific dose of DEHP reached significance using Dunnett's *post hoc* test. There were no significant changes in blood glucose. Of note, there was a decrease trend in glucose levels in the 300 mg DEHP per kg per day (*P*=0.0668; Figure 5).

## Discussion

The incidence of the metabolic syndrome has dramatically increased over the past 25 years. Criteria for a clinical diagnosis of metabolic syndrome consist of an increase in waist circumference, triglycerides, blood pressure, fasting glucose and reduced high-density lipoprotein levels.^29^ Increased abdominal obesity and sedentary lifestyles are considered major risk factors.^29^ Many factors may be responsible for increased obesity, including exposure to endocrine disruptors, which are now considered obesogenic compounds.^30^ Indeed, it was suggested that these chemicals might be defined as metabolic disruptors because they affect metabolic signaling.^31^

Numerous mechanisms may underlie endocrine disruptor effects on non-developmental obesity (including PPAR pathway activation and perturbation of thyroid function) and *in utero* exposure.^30^ It has been proposed that exposure to endocrine disruptors during fetal life causes permanent metabolic alterations,^32, 33^ possibly through epigenetic mechanisms,^34^ which result in development of obesity later in life.^32, 33^ Evidence supporting this hypothesis was observed by Schmidt *et al.*^33^ in their findings that exposing mice to a diet containing low doses (0.05 and 5 mg DEHP per kg per day, corresponding to a daily intake of 0.00102 and 0.108 DEHP per kg per day) of DEHP *in utero* and during lactation resulted in increased visceral fat and body weight in adult offspring. In agreement with previous work in rats,^13, 35^ we did not observe changes in total body weight. It remains to be determined whether a differential fat distribution is induced by DEHP without modifying the total weight of the animals and whether more drastic changes would develop later in time during senescence.

Using the DAVID bioinformatics portal, we observed that DEHP targeted the PPAR pathway and several metabolic pathways in the SVF and immune-related pathways in whole-adipose tissue. The PPAR pathway changes were downstream of the three known PPARs and associated with fatty acid metabolism, bile acid biosynthesis, synthesis and degradation of ketone bodies, glycerophospholipid metabolism and gluconeogenesis. MEHP is known to activate PPARα and PPARγ nuclear receptors.^36^

We report increased gene expression of *RT1-Bb* and *RT1-EC16* in whole-adipose tissue and of *Ccr1*, *Cxcr4* and *Tnf* in the SVF. These markers are associated with antigen processing and presentation and cytokine–cytokine receptor interaction in immune cells. Similar increases in inflammatory marker expression have been reported after DEHP and MEHP treatment in different monocyte/macrophage models, such as the macrophage RAW264.7 cell line,^37^ human acute monocytic leukemia THP-1 cell line^38^ and rat alveolar macrophages.^39^ Moreover, MEHP increases IL-5 and IL-10 levels in cultures of local lymph node cells, suggesting that phthalates activate the adaptive immune system.^40^ Taken together, these data suggest that *in utero* DEHP exposure induces an inflammatory state in the adult adipose tissue of male offspring.

We observed increased serum TNF levels in animals exposed to 300 mg DEHP per kg per day, suggesting chronic low-grade inflammation caused by *in utero* DEHP exposure. We localized TNF production to the SVF, where *Tnf* mRNA was upregulated. Quantification of TNF levels in whole-adipose lyses showed increased local TNF production starting at the 50 mg kg^−1^ per day *in utero* exposure dose, which correlates to the increased serum TNF levels, although not significant. TNF is a cytokine involved in inflammation and host-defense responses,^41^ and is primarily produced by macrophages and monocytes, which are abundant in the SVF.^42^ Other low-level sources of TNF include cardiac myocytes, keratinocytes^41^ and adipocytes.^43^ Further histological analyses showed focal adipose macrophage presence that correlates with the increased serum TNF levels. Interestingly, exposure to 1 mg DEHP per kg per day was sufficient to increase the monocyte/macrophage marker CD163, thus revealing the monocyte/macrophages as a sensitive target of DEHP. The increased TNF and CD163 expression on the contrary was not seen at 20 mg DEHP per kg per day; this could be due to the phenomenon of hormesis,^44^ that is, small and large concentrations of a substance, including the phthalates,^45^ have opposite effects on a cell and tissue function, and subsequent outcomes. Although the 300 mg DEHP per kg per day dose was the one with significant effects, for several parameters such as some adipose and SVF genes, and the aforementioned adipose tissue TNF and CD163 levels, there was an apparent dose–response effect. Finally, it is interesting to notice that also the TNF serum levels showed an increasing dose–response trend, although we did not reach significance. All these data together suggest that the observed phenotype might really be a direct effect of DEHP on the offspring organogenesis and physiology.

The role of TNF in metabolism has been observed in knockout models, where TNF reduction improves insulin sensitivity by increasing peripheral glucose uptake.^43, 46^ These results suggest that fetal exposure to low doses of DEHP induces macrophage infiltration in the adult adipose tissue, which increases local production of TNF and thus increases serum TNF levels.

A main function of TNF is to induce liver production of CRP via the IL-6/IL-1 pathway.^41^ CRP is an acute phase inflammation protein whose levels sharply increase (up to 1000-fold) during the inflammatory process.^41^ We observed a significant increase in serum CRP levels in animals exposed to 300 mg DEHP per kg per day. Interestingly, neither liver and adipose *Crp* mRNA levels nor adipose tissue lysate CRP levels were affected by DEHP, suggesting another source accounts for this increase. Additional CRP production has been reported in many organs such as adipose tissue,^47^ kidney^48^ and pancreas.^49^ These data suggest that DEHP targeted an unidentified tissue resulting in increased CRP levels that, together with TNF, contributed to a low-grade inflammatory state. Low-grade inflammation is believed to have a role in the development of obesity and metabolic syndrome.^41^ In our study, IL-6 was not increased in the epididymal adipose tissue but it was significantly modified in retroperitoneal tissue at the dose of 1 mg DEHP per kg per day. Increased retroperitoneal tissue levels of TNF were seen, suggesting a differential tissue-specific response to DEHP. It remains to be examined whether DEHP-induced TNF and IL-6 is associated with CRP levels or is a consequence of the effects of DEHP in other target tissues. CRP, TNF and IL-6 have been positively correlated with visceral adiposity.^50^ In addition to our data showing changes at 1 mg DEHP per kg per day in epididymal and retroperitoneal adipose tissue, we also reported that DEHP targeted the PPAR pathway in the adrenal gland starting at 1 mg DEHP per kg per day.^51^ Taken all together, the data suggest that DEHP promotes multi-organ changes in gene expression and inflammatory markers at doses relevant for human exposure.

Pathway analyses also identified RAS components that were up- and downregulated by DEHP in whole-adipose tissue and the SVF. The pathway studies revealed that DEHP upregulated enzymes associated with conversion of AT I to the more active ATII in whole-adipose tissue. In the SVF, angiotensinogen and the ATII receptors were downregulated, suggesting that a feedback loop was triggered in response to the local AII increase. Human adipose tissue expresses all components of the RAS pathway^52^ and its deregulation has been associated with obesity.^53^ A link between RAS and inflammation has also been established using transgenic mice overexpressing angiotensinogen in adipose tissue, which increased TNF, IL-6 and IL-1β mRNA levels.^54^ Moreover, TNF was shown to modulate adipose RAS function by increasing ATII production in isolated subcutaneous adipocytes.^55^ Mice that overexpress angiotensinogen in adipose tissue have increased circulating angiotensinogen levels and body mass, and are hypertensive.^56^ Our previous study showed that DEHP targeted AT1 and AT2 receptors in the adrenal gland without increasing angiotensinogen mRNA levels in the liver or circulating levels of AT II.^16^ These observations, together with the finding of decreased blood pressure in elderly rats exposed *in utero* to DEHP,^57^ suggest that DEHP targets the local adipose RAS pathway without affecting systemic RAS. In humans, similar correlations between RAS and obesity have been found. Increases in the mRNA expression of renin, AT-converting enzyme and ATR1 in adipose tissue have been reported in an obese and hypertensive cohort.^58^ The relationship between RAS and obesity appears to be dynamic because 5% reductions of body weight in postmenopausal woman decreased components of the RAS pathway in plasma and adipose tissue.^59^

We identified five different fatty acid-binding proteins that were upregulated by DEHP in global gene expression analyses, but only *Fabp4* was significantly upregulated using quantitative reverse transcription-PCR . Fatty acid-binding proteins are a family of highly expressed intracellular lipid-binding proteins that traffic fatty acids and their derivatives throughout cellular compartments (for example, peroxisomes, mitochondria and lipid droplets).^60^ Ten different genes encoding fatty acid-binding protein isoforms have been identified in mammals.^60^ Fatty acid-binding protein-4 (AP2) is highly expressed in monocytes and macrophages, and is found in white and brown adipose tissue, where it serves as a differentiation marker for adipogenesis. Increased expression of *Fabp4*, together with increased expression of the differentiation marker *Cebpa*,^61^ suggests that DEHP may increase the rate or capacity of pre-adipocyte differentiation.

We hypothesize that epigenetic modifications that occur during *in utero* DEHP exposure are driving the changes observed in the adult offspring. Pre-adipocyte development initiates around gestational day 15^62^ and coincides with the window of DEHP treatment, which might result in faulty pre-adipocyte development. However, the trigger that initiates adipose tissue macrophage infiltration and increased CRP serum levels remains to be determined.

In conclusion, *in utero* DEHP exposure promotes local inflammation in adipose tissue and low-grade chronic systemic inflammation in the adult offspring. We identified macrophages as a sensitive target of DEHP and the likely source of TNF production. We report evidence suggesting that DEHP affects pre-adipocyte development causing metabolically hindered pre-adipocytes, which ultimately leads to deregulation of the local RAS system. We hypothesize that disruption of pre-adipocyte development due to *in utero* DEHP exposure is a significant risk factor for the development of metabolic syndrome in adult offspring.

## Figures and Tables

**Table 1:** Primer sequences used in qRT-PCR analyses

*Gene*	*Forward primer (5′–3′)*	*Reverse primer (5′–3′)*	*Annealing*	*Gene ID*
*Actb*	CGCAGACACTTTCTACAATGAGCTGCG	ACGGTTGGCCTTAGGGTTCAG	61 °C	81822
*Ccr1*	CCGTGCGTCTGATATTTGC	GTCAGATTGTAGGGGGTCCA	60 °C	57301
*Cebpa*	CGGTAAAGAACAGCAACGAGTACCG	GCGCTGTTTGGCTTTATCTCG	61 °C	24252
*Cma1*	GGATGAATCTCCATGCTCTGT	CGATGATCTCCCCAGCTTT	60 °C	25627
*Cpa3*	AAACCGTTCCAAGAACCCAAAT	CGATGTGGAAGAGTCCCATGAGACATCG	61 °C	54242
*Crp*	GGACAAATGCAAGCATCATC	AGACTGATTCGCGTCAAAGC	60 °C	25419
*Cxcr4*	CGTCTATGTGGGTGTCTGGA	GCGAAGATGATGTCAGGGATA	60 °C	60628
*Fabp4*	CGAAGTAACTCGTCTCCAGTGAGAACTTCG	CCATACCGGCCACTTTCCTG	61 °C	79451
*Lgals3bp*	CGACTCAATGCACAGGGAACGAGTCG	GTCCTTCTCATGCCCGCAGT	61 °C	245955
*Mcpt8*	AACCCAGGTCATCGCTGTTG	CGGTTGGAGCATGATGTCATTAAACCG	61 °C	29269
*Mcpt9*	CGTGATCTAAAGGCCAAACCTCACG	TTGAGCTTTGCGTTCCAACTTC	61 °C	54272
*Mcpt10*	GGCCATCCTACCAGTCAACACT	CGGCTATGAATGCCATGTAGGGCCG	61 °C	54269
*RT1-Bb*	GACAGATTTCTACCCAGCCCAGA	CGTGTCTTCCTAATAAGCTGTGTGGACACG	61 °C	309622
*RT1-EC16*	AGGGCAAGTGCTTGGAGTC	CCTTTGGGGGATCTGAGC	60 °C	414819
*Tnf*	CCCTGGTACTAACTCCCAGAAA	TGTATGAGAGGGACGGAACC	60 °C	24835

Abbreviations: Actb, β-actin; Ccr1, chemokine (C–C motif) receptor 1; Cebpa, CCAAT/enhancer-binding protein α Cma1, chymase 1; Cpa3, carboxypeptidase A3; Crp, C-reactive protein; Cxcr4, C-X-C chemokine receptor type 4; Fabp4, fatty-acid-binding protein 4; Lgals3bp, galactoside-binding, soluble, 3 binding protein; Mcpt8, mast cell protease 8; Mcpt9, mast cell protease 9; Mcpt10, mast cell protease 10; qRT-PCR, quantitative reverse transcription-PCR; RT1-Bb, RT1 class II, locus Bb; RT1-EC16, RT1 class I locus EC16; Tnf, tumor necrosis factor.
