# Supplementary material for: In utero exposure to the endocrine disruptor di-(2-ethylhexyl) phthalate promotes local adipose and systemic inflammation in adult male offspring
Source: Nutr Diabetes. 2014 May 5;4(5):e115–. doi: 10.1038/nutd.2014.13 (PMC4042311; doi:10.1038/nutd.2014.13)
Supplement: Supplementary Tables [file nutd201413x1.doc]

**Table S1. Significantly changed genes in the SVF**

| **Probe Set ID** | **Fold change** | **Gene Symbol** | **Chromosomal Location** |
| --- | --- | --- | --- |
| 1393302_at | 4.93 | --- | --- |
| 1375212_at | 3.77 | Ankrd52 | chr7q11 |
| 1388056_at | 3.38 | Oas1b | chr12q16 |
| 1394534_at | 2.62 | --- | --- |
| 1368437_at | 2.57 | Car4 | chr10q26 |
| 1368826_at | 2.50 | Comt | chr11q23 |
| 1382845_at | 2.50 | --- | --- |
| 1397769_at | 2.48 | --- | --- |
| 1382257_at | 2.44 | --- | --- |
| 1391524_at | 2.21 | RGD1564964 | chr8q31 |
| 1369889_at | 2.17 | Ifnb1 | chr5q31-q33 |
| 1385465_at | 2.15 | Siglec5 | chr1q22 |
| 1368630_at | 2.11 | Fabp9 | chr2q23 |
| 1391895_at | 2.09 | --- | --- |
| 1390310_at | 2.06 | Icam2 | chr10q32.1 |
| 1379357_at | 2.02 | --- | --- |
| 1369724_at | 1.96 | F13a1 | chr17p12 |
| 1394683_at | 1.96 | --- | --- |
| 1381386_at | 1.95 | Pop5 | chr12q16 |
| 1387712_at | 1.92 | Hes3 | chr5q36 |
| 1391579_at | 1.91 | --- | --- |
| 1380445_at | 1.91 | --- | --- |
| 1391949_at | 1.88 | --- | --- |
| 1384985_at | 1.88 | --- | --- |
| 1386769_at | 1.88 | --- | --- |
| 1390041_at | 1.86 | Sox17 | chr5q12 |
| 1378074_at | 1.86 | --- | --- |
| 1370150_a_at | 1.85 | Thrsp | chr1q32 |
| 1369497_at | 1.85 | LOC24906 | chr10q22 |
| 1389119_at | 1.84 | Xirp1 | chr8q32 |
| 1371400_at | 1.83 | Thrsp | chr1q32 |
| 1377632_at | 1.83 | Timp4 | chr4q42 |
| 1378003_at | 1.81 | Lrrc8b | chr14p22 |
| 1374779_at | 1.81 | F13a1 | chr17p12 |
| 1373259_at | 1.80 | --- | --- |
| 1380531_at | 1.80 | Slc9a9 | chr8q31 |
| 1393525_at | 1.80 | --- | --- |
| 1393751_at | 1.80 | Fabp12 | chr2q23 |
| 1387852_at | 1.79 | Thrsp | chr1q32 |
| 1398507_at | 1.78 | --- | --- |
| 1389503_at | 1.77 | LOC367994 | chr12q12 |
| 1367552_at | 1.76 | Svs4 | chr3q42 |
| 1379324_at | 1.76 | --- | --- |
| 1378546_at | 1.75 | --- | --- |
| 1390956_at | 1.75 | Apol11a | chr7q34 |
| 1368683_at | 1.73 | Olr1 | chr4q42 |
| 1391600_at | 1.73 | Mga | chr3q35 |
| 1391500_at | 1.73 | --- | --- |
| 1381679_at | 1.72 | --- | --- |
| 1385599_at | 1.72 | RSA-14-44 | chr4q31 |
| 1391095_at | 1.71 | Mmp19 | chr7q11 |
| 1393927_at | 1.71 | Wnt2 | chr4q21-q22 |
| 1392308_at | 1.71 | Pla2g2d | chr5q36 |
| 1374017_at | 1.70 | Trim72 | chr1q36 |
| 1397320_at | 1.70 | --- | --- |
| 1396933_s_at | 1.70 | Akr1c14 | chr17q12.3 |
| 1396249_at | 1.70 | --- | --- |
| 1385201_at | 1.70 | Tmem182 | chr9q21 |
| 1387168_at | 1.68 | Cd93 | chr3q41 |
| 1369345_at | 1.68 | Inpp4b | chr19q11 |
| 1371082_at | 1.68 | Arr3 | chrXq31 |
| 1377366_at | 1.68 | --- | --- |
| 1391977_at | 1.68 | --- | --- |
| 1368317_at | 1.67 | Aqp7 | chr5q22 |
| 1368892_at | 1.67 | --- | --- |
| 1381971_at | 1.67 | Sox18 | chr3q43 |
| 1375066_at | 1.67 | RGD1563319 | chr1q41 |
| 1391384_at | 1.67 | Tnf | chr20p12 |
| 1379845_at | 1.67 | --- | --- |
| 1368975_at | 1.66 | Cd38 | chr14q21 |
| 1384670_at | 1.65 | Dnajc17 | chr3q35 |
| 1384328_at | 1.65 | Tom1 | chr19p11 |
| 1383189_at | 1.64 | --- | --- |
| 1368407_at | 1.64 | Hpse | chr14p22 |
| 1390971_at | 1.64 | --- | --- |
| 1367975_at | 1.64 | Anxa3 | chr14p22 |
| 1387589_at | 1.63 | Rims3 | chr5q36 |
| 1381575_at | 1.63 | Neb | chr3q12 |
| 1391587_at | 1.63 | --- | --- |
| 1383032_at | 1.62 | --- | --- |
| 1385096_at | 1.62 | --- | --- |
| 1369405_a_at | 1.62 | Chrnb4 | chr8q24 |
| 1377711_at | 1.62 | --- | --- |
| 1381729_at | 1.62 | LOC498675 | --- |
| 1382936_at | 1.62 | --- | --- |
| 1376510_at | 1.62 | --- | --- |
| 1370083_at | 1.61 | Ccr1 | chr8q32 |
| 1393411_at | 1.60 | Cfp | chrXq12 |
| 1386189_at | 1.60 | --- | --- |
| 1378220_at | 1.60 | LOC497860 | chr10q11 |
| 1376675_at | 1.60 | --- | --- |
| 1387825_at | 1.60 | Ugt2b | chr14p22-p21.2 |
| 1390325_at | 1.60 | --- | --- |
| 1391002_at | 1.60 | --- | --- |
| 1384702_at | 1.60 | --- | --- |
| 1397944_at | 1.60 | --- | --- |
| 1368358_a_at | 1.60 | Ptprr | chr7q22 |
| 1385768_at | 1.59 | --- | --- |
| 1391132_at | 1.59 | Car4 | chr10q26 |
| 1383906_at | 1.59 | Neurl3 | chr9q21 |
| 1368473_at | 1.59 | Gja5 | chr2q34 |
| 1374140_at | 1.59 | --- | --- |
| 1383644_at | 1.58 | --- | --- |
| 1370708_a_at | 1.58 | Akr1c14 | chr17q12.3 |
| 1385822_at | 1.58 | --- | --- |
| 1376968_at | 1.58 | Mybpc2 | chr1q22 |
| 1371172_at | 1.58 | Atp2b3 | chrXq37 |
| 1382966_at | 1.58 | --- | --- |
| 1396862_at | 1.58 | --- | --- |
| 1387993_at | 1.58 | Cyp2b21 | chr1q21 |
| 1395649_at | 1.58 | Lhfpl4 | chr4q42 |
| 1384862_at | 1.57 | --- | --- |
| 1374801_at | 1.57 | --- | --- |
| 1368988_at | 1.56 | Casq2 | chr2q34 |
| 1369389_at | 1.56 | Znf483 | chr5q24 |
| 1378386_at | 1.56 | Slc2a6 | chr3p12 |
| 1389645_at | 1.56 | Prodh2 | chr1q21 |
| 1378797_at | 1.56 | --- | --- |
| 1370432_at | 1.56 | Pou3f1 | chr5q36 |
| 1375862_at | 1.56 | Pxdn | chr6q16 |
| 1384074_at | 1.56 | --- | --- |
| 1381311_at | 1.55 | Emr1 | chr9q11 |
| 1380700_at | 1.55 | Arhgap27 | chr10q32.1 |
| 1375924_at | 1.55 | --- | --- |
| 1397461_at | 1.55 | Glt8d2 | chr7q13 |
| 1386936_at | 1.55 | Grifin | chr12q11 |
| 1377765_at | 1.55 | Clic4 | chr5q36 |
| 1392413_at | 1.55 | --- | --- |
| 1384300_at | 1.55 | --- | --- |
| 1368704_a_at | 1.54 | Cspg5 | chr8q32.1 |
| 1379248_at | 1.54 | Prcp | chr1q32 |
| 1381649_at | 1.54 | --- | --- |
| 1394876_at | 1.53 | --- | --- |
| 1388240_a_at | 1.53 | Itga7 | chr7q11 |
| 1375952_at | 1.53 | --- | --- |
| 1391128_at | 1.53 | Bcl9l | chr8q22 |
| 1393765_at | 1.53 | --- | --- |
| 1398726_at | 1.53 | Ascc3l1 | chr3q36 |
| 1381753_at | 1.53 | --- | --- |
| 1381298_at | 1.53 | --- | --- |
| 1373872_at | 1.52 | Mgat4a | chr9q21 |
| 1382106_at | 1.52 | Ccl6 | chr10q26 |
| 1382078_at | 1.52 | --- | --- |
| 1380484_at | 1.52 | Pi16 | chr20p12 |
| 1390348_at | 1.52 | Folr2 | chr1q32 |
| 1388891_at | 1.52 | Parvb | chr7q34 |
| 1368534_at | 1.52 | Adra1d | chr3q36 |
| 1391071_at | 1.52 | --- | --- |
| 1391442_at | 1.52 | Ehd3 | chr6q13 |
| 1392349_at | 1.52 | Slc5a3 | chr11q11 |
| 1397610_at | 1.52 | --- | --- |
| 1373661_a_at | 1.52 | Cxcr4 | chr13q12 |
| 1379820_at | 1.52 | --- | --- |
| 1392052_at | 1.52 | --- | --- |
| 1394200_at | 1.52 | Hspa2 | chr6q24 |
| 1370176_at | 1.52 | Trak2 | chr9q31 |
| 1383412_at | 1.52 | --- | --- |
| 1369694_at | 1.52 | Slc1a2 | chr3q31 |
| 1389548_at | 1.52 | Adhfe1 | chr5q11 |
| 1382192_at | 1.52 | Lyve1 | chr1q33 |
| 1385798_at | 1.51 | --- | --- |
| 1367700_at | 1.51 | Fmod | chr13q13 |
| 1396513_at | 1.51 | --- | --- |
| 1384262_at | 1.51 | Ppp1r3b | chr16q12.2 |
| 1395078_at | 1.51 | Sema3g | chr16p16 |
| 1389553_at | 1.51 | Clec4a3 | chr4q42 |
| 1390849_at | 1.51 | --- | --- |
| 1370080_at | 1.51 | Hmox1 | chr19p11 |
| 1387687_at | 1.51 | Igsf6 | chr1q36 |
| 1369003_at | 1.51 | Dedd | chr13q24 |
| 1369273_a_at | 1.51 | Npr3 | chr2q16 |
| 1393235_at | 1.51 | --- | --- |
| 1391574_at | 1.50 | --- | --- |
| 1398703_at | 1.50 | --- | --- |
| 1369425_at | 1.50 | Cdh13 | chr19q12 |
| 1385381_at | 1.50 | --- | --- |
| 1391856_at | 1.50 | Sema3g | chr16p16 |
| 1395086_at | 1.50 | Patl1 | chr1q43 |
| 1379818_at | 1.50 | --- | --- |
| 1391424_at | 1.50 | --- | --- |
| 1369456_at | 1.50 | Htr2b | chr9q35 |
| 1371910_at | -1.50 | --- | --- |
| 1367740_at | -1.50 | Ckb | chr6q32 |
| 1397419_at | -1.50 | Mpp6 | chr4q24 |
| 1375646_at | -1.50 | Efcab2 | chr13q26 |
| 1396815_at | -1.50 | --- | --- |
| 1380719_at | -1.50 | --- | --- |
| 1373321_at | -1.50 | RGD1306622 | chr1q43 |
| 1383765_at | -1.50 | Dmrtc1a | --- |
| 1386466_at | -1.50 | --- | --- |
| 1383309_at | -1.50 | St3gal6 | chr11q12 |
| 1382318_at | -1.50 | --- | --- |
| 1389354_at | -1.50 | Gipc2 | chr2q45 |
| 1368035_a_at | -1.50 | Ptprf | chr5q36 |
| 1368453_at | -1.51 | Fads2 | chr1q43 |
| 1387058_at | -1.51 | Pctp | chr10q26 |
| 1369161_at | -1.51 | Abcb4 | chr4q11-q12 |
| 1390986_at | -1.51 | --- | --- |
| 1370459_at | -1.51 | Aard | chr7q31 |
| 1387811_at | -1.51 | Agt | chr19q12 |
| 1374743_at | -1.51 | Inadl2 | chr5q33 |
| 1381445_at | -1.51 | --- | --- |
| 1398444_at | -1.51 | RGD1311742 | chr18p11 |
| 1397744_at | -1.51 | --- | --- |
| 1376009_at | -1.51 | --- | --- |
| 1367647_at | -1.51 | Serpina1 | chr6q32 |
| 1390292_at | -1.51 | Tmem8a | chr10q12 |
| 1368114_at | -1.51 | Fgf13 | chrXq36 |
| 1374190_at | -1.51 | Clybl | chr15q25 |
| 1393058_at | -1.52 | Eid2 | chr1q21 |
| 1368092_at | -1.52 | Fah | chr1q31 |
| 1385707_at | -1.52 | Lect2 | chr17p14 |
| 1393491_at | -1.52 | Tbl1x | chrXq22 |
| 1387821_at | -1.52 | Rab3ip | chr7q22 |
| 1378523_at | -1.52 | Ttc12 | chr8q23 |
| 1393696_at | -1.52 | Fibin | chr3q33 |
| 1368676_at | -1.52 | Dync2h1 | chr8q11 |
| 1374493_at | -1.52 | --- | --- |
| 1378034_at | -1.52 | Mterfd3 | chr7q13 |
| 1395471_at | -1.52 | --- | --- |
| 1387394_at | -1.52 | Il2rb | --- |
| 1377264_at | -1.52 | Il17f | chr9q13 |
| 1390483_at | -1.53 | Slc25a29 | chr6q32 |
| 1368202_a_at | -1.53 | Dab2 | chr2q16 |
| 1368057_at | -1.53 | Abcd3 | chr2q41 |
| 1378720_at | -1.53 | Gli1 | chr7q22 |
| 1368144_at | -1.53 | Rgs2 | chr13q21 |
| 1371347_at | -1.53 | Tmem50b | chr11q11 |
| 1370486_a_at | -1.53 | Iiig9 | chr1q43 |
| 1383487_at | -1.53 | --- | --- |
| 1377558_at | -1.53 | --- | --- |
| 1389774_at | -1.53 | Znf23 | chr19q12 |
| 1386160_at | -1.53 | Tchh | chr2q34 |
| 1384554_at | -1.53 | --- | --- |
| 1388963_at | -1.53 | Astn1 | chr13q22 |
| 1376197_at | -1.54 | --- | --- |
| 1392055_at | -1.54 | --- | --- |
| 1380981_at | -1.54 | Ston2 | chr6q31 |
| 1376222_at | -1.54 | Sdr39u1 | chr15p13 |
| 1377469_at | -1.54 | --- | --- |
| 1385043_at | -1.54 | --- | --- |
| 1371951_at | -1.54 | Fhl2 | chr9q22 |
| 1380245_at | -1.54 | --- | --- |
| 1375621_at | -1.54 | Sfxn5 | chr4q34 |
| 1392541_at | -1.54 | Ggct | chr4q24 |
| 1377753_at | -1.55 | --- | --- |
| 1392864_at | -1.55 | Arhgap5 | chr6q23 |
| 1383870_at | -1.55 | Hoxb6 | chr10q31 |
| 1370848_at | -1.55 | Slc2a1 | chr5q36.1 |
| 1391269_at | -1.55 | Pim2 | chrXq13 |
| 1378106_at | -1.55 | Phlda2 | chr1q41 |
| 1374865_at | -1.55 | RGD1562618 | chr8q24 |
| 1378566_at | -1.55 | --- | --- |
| 1389412_at | -1.55 | --- | --- |
| 1397167_at | -1.55 | --- | --- |
| 1371889_at | -1.55 | Slc22a17 | chr15p13 |
| 1375959_at | -1.55 | Nkd1 | chr19p11 |
| 1390649_at | -1.55 | Slc30a3 | chr6q14 |
| 1368253_at | -1.56 | Gamt | chr7q11 |
| 1394570_at | -1.56 | Xk | chrXq13 |
| 1395948_at | -1.56 | --- | --- |
| 1392550_at | -1.56 | Dact1 | chr6q24 |
| 1371298_at | -1.56 | H19 | chr1q41 |
| 1393712_at | -1.56 | --- | --- |
| 1382387_at | -1.56 | Ano1 | chr1q42 |
| 1387111_at | -1.56 | Ddah1 | chr2q44 |
| 1383324_at | -1.56 | Mpp6 | chr4q24 |
| 1389782_at | -1.56 | RGD1305587 | chr20q11 |
| 1368455_at | -1.56 | Nkg7 | chr1q22 |
| 1383869_at | -1.56 | --- | --- |
| 1386976_at | -1.56 | Cd82 | chr3q24 |
| 1368641_at | -1.56 | Wnt4 | chr5q36 |
| 1375016_at | -1.56 | --- | --- |
| 1385072_at | -1.56 | Galm | chr6q11 |
| 1383442_at | -1.56 | Morn1 | chr5q36 |
| 1368081_at | -1.56 | Abca2 | chr3p13 |
| 1393474_at | -1.56 | Sult2b1 | chr1q22 |
| 1387970_at | -1.56 | Slc38a5 | chrXq13 |
| 1390906_at | -1.56 | --- | --- |
| 1388773_at | -1.57 | Tnfaip2 | chr6q32 |
| 1377060_at | -1.57 | Mccc2 | chr2q12 |
| 1368368_a_at | -1.57 | Lsr | chr1q21 |
| 1380834_at | -1.57 | Btnl8 | chr20p12 |
| 1382090_at | -1.57 | --- | --- |
| 1377663_at | -1.57 | Rnd3 | chr3q12 |
| 1376311_at | -1.57 | Ntng1 | chr2q41 |
| 1385791_at | -1.57 | Vill | chr8q32 |
| 1370291_at | -1.57 | Pdlim3 | chr16q11 |
| 1395858_at | -1.57 | --- | --- |
| 1384315_at | -1.57 | Pdk3 | chrXq22 |
| 1376668_at | -1.57 | Ttc39a | chr5q35 |
| 1370394_at | -1.57 | IgG-2a | chr6q32 |
| 1390857_at | -1.57 | Xylb | chr8q32 |
| 1369306_at | -1.58 | Klrd1 | chr4q42 |
| 1381125_at | -1.58 | --- | --- |
| 1373337_at | -1.58 | Grhpr | chr5q22 |
| 1395446_at | -1.58 | --- | --- |
| 1383783_at | -1.58 | Pcdh9 | chr15q21 |
| 1373975_at | -1.58 | Inmt | chr4q24 |
| 1377412_at | -1.58 | --- | --- |
| 1383915_at | -1.58 | LOC686120 | chr12q12 |
| 1383355_at | -1.58 | Abca1 | chr5q24 |
| 1379592_at | -1.58 | Slc25a13 | chr4q13 |
| 1387271_at | -1.58 | Phyh | chr17q12.3 |
| 1381163_at | -1.58 | --- | --- |
| 1395209_at | -1.58 | --- | --- |
| 1377457_a_at | -1.58 | Sorl1 | chr8q22 |
| 1381858_at | -1.59 | Trim14 | chr5q22 |
| 1380479_at | -1.59 | --- | --- |
| 1398255_at | -1.59 | Slc15a2 | chr11q22 |
| 1372929_at | -1.59 | Kcnma1 | chr15p16 |
| 1368870_at | -1.59 | Id2 | chr6q16 |
| 1371997_at | -1.59 | Akr1cl2 | chr17q12.3 |
| 1390710_x_at | -1.59 | Sorl1 | chr8q22 |
| 1372325_at | -1.59 | Emilin1 | chr6q14 |
| 1375199_at | -1.59 | --- | --- |
| 1383435_at | -1.59 | Scn3b | chr8q22 |
| 1379052_at | -1.59 | --- | --- |
| 1391302_at | -1.59 | --- | --- |
| 1369313_at | -1.59 | Fhl2 | chr9q22 |
| 1370330_at | -1.59 | Sipa1l1 | chr6q24 |
| 1385724_at | -1.59 | Ccdc74a | chr11q23 |
| 1368131_at | -1.59 | Capn6 | chrXq14 |
| 1369318_at | -1.59 | Fhit | chr15p15-p14 |
| 1392053_at | -1.60 | Mmrn1 | chr4q24 |
| 1382729_at | -1.60 | RGD1564019 | chr3p11 |
| 1389521_at | -1.60 | Ivns1abp | chr13q21 |
| 1375553_at | -1.60 | RGD1311742 | chr18p11 |
| 1371916_at | -1.60 | Sepx1 | chr10q12 |
| 1382685_at | -1.60 | Slit2 | chr14q11 |
| 1367707_at | -1.60 | Fasn | --- |
| 1390036_at | -1.60 | Slc16a6 | chr10q32.1 |
| 1397267_at | -1.60 | Rimbp2 | chr12q14 |
| 1398378_at | -1.60 | Gstk1 | chr4q23 |
| 1368013_at | -1.60 | Ddit4l | chr2q44 |
| 1393020_at | -1.61 | LOC100361631 | --- |
| 1380547_at | -1.61 | Clcn3 | chr16p12 |
| 1377220_at | -1.61 | --- | --- |
| 1384500_at | -1.61 | Ccdc30 | chr5q36 |
| 1381937_at | -1.61 | --- | --- |
| 1383645_at | -1.61 | --- | --- |
| 1389562_at | -1.61 | --- | --- |
| 1397646_at | -1.61 | --- | --- |
| 1382197_at | -1.62 | Rhod | chr1q42 |
| 1392126_at | -1.62 | --- | --- |
| 1383153_at | -1.62 | --- | --- |
| 1368716_at | -1.62 | Ppp1r14c | chr1p11 |
| 1397900_at | -1.62 | --- | --- |
| 1392823_at | -1.62 | Snhg11 | chr3q42 |
| 1368477_at | -1.62 | Atp2a3 | chr10q24 |
| 1377009_at | -1.62 | --- | --- |
| 1383722_at | -1.62 | Prosc | chr16q12.4 |
| 1375958_at | -1.62 | --- | --- |
| 1376790_at | -1.62 | --- | --- |
| 1379776_at | -1.62 | --- | --- |
| 1376537_at | -1.62 | Ptpn3 | chr5q24 |
| 1380836_at | -1.63 | RGD1562726 | chr11q11 |
| 1379057_at | -1.63 | LOC683460 | --- |
| 1392118_at | -1.63 | --- | --- |
| 1367835_at | -1.63 | Pcsk1n | chrXq13 |
| 1375873_at | -1.63 | RGD1561149 | chr5q36 |
| 1384264_at | -1.63 | Myh14 | chr1q22 |
| 1391011_at | -1.63 | --- | --- |
| 1377513_at | -1.63 | --- | --- |
| 1379814_at | -1.63 | --- | --- |
| 1392521_at | -1.63 | --- | --- |
| 1374892_at | -1.63 | Sat2 | chr10q24 |
| 1386917_at | -1.63 | Pc | chr1q42 |
| 1390050_at | -1.63 | LOC680692 | chr17p14 |
| 1378301_at | -1.63 | --- | --- |
| 1383422_at | -1.64 | Bend5 | chr5q35 |
| 1380886_at | -1.64 | --- | --- |
| 1384143_at | -1.64 | Cgn | chr2q34 |
| 1389411_at | -1.64 | --- | --- |
| 1376161_at | -1.64 | Tmed4 | chr14q21 |
| 1385837_at | -1.64 | Hoxd3 | chr3q23 |
| 1393130_at | -1.64 | --- | --- |
| 1392062_a_at | -1.64 | Hexdc | --- |
| 1377914_at | -1.64 | Srrm1 | chr5q36 |
| 1394968_at | -1.64 | --- | --- |
| 1368333_at | -1.65 | Umod | chr1q36-q37 |
| 1395991_at | -1.65 | Rimbp2 | chr12q14 |
| 1398528_at | -1.65 | --- | --- |
| 1370104_at | -1.65 | Pde6h | chr4q43 |
| 1375900_at | -1.65 | Tnfrsf9 | chr5q36 |
| 1375908_at | -1.65 | Mpzl2 | chr8q22 |
| 1383946_at | -1.65 | Cldn1 | chr11q22 |
| 1384531_at | -1.65 | Crb3 | chr9q11 |
| 1369292_at | -1.65 | Hsd17b1 | chr10q32.1 |
| 1369609_at | -1.65 | Cldn11 | chr2q24 |
| 1371942_at | -1.65 | Gstt3 | chr20p12 |
| 1369531_at | -1.66 | Sult1c2 | chr9q11 |
| 1377283_at | -1.66 | Hps4 | chr19p11 |
| 1367632_at | -1.66 | Glul | chr13q22 |
| 1386718_at | -1.66 | Akr1c19 | chr17q12.3 |
| 1393605_at | -1.66 | Vav3 | chr2q34-q41 |
| 1380343_at | -1.66 | Thada | chr6q12 |
| 1378269_at | -1.66 | Dnal1 | chr6q31 |
| 1384936_at | -1.66 | --- | --- |
| 1368689_at | -1.66 | Gjb5 | chr5q36 |
| 1378434_at | -1.67 | --- | --- |
| 1384696_at | -1.67 | --- | --- |
| 1396179_at | -1.67 | --- | --- |
| 1374738_at | -1.67 | Sdccag10 | chr2q13 |
| 1395652_at | -1.67 | Gyltl1b | chr3q24 |
| 1368470_at | -1.67 | Ggh | chr5q21 |
| 1376217_at | -1.67 | Casc1 | chr4q44 |
| 1381841_at | -1.67 | --- | --- |
| 1387945_at | -1.67 | LOC681290 | chr17q11 |
| 1369470_at | -1.67 | Akap14 | chrXq12 |
| 1372962_at | -1.67 | Tarbp2 | chr7q36 |
| 1396123_at | -1.68 | Tctex1d2 | chr11q22 |
| 1377015_at | -1.68 | --- | --- |
| 1371084_at | -1.68 | Klk1c10l2 | chr1q22 |
| 1373312_at | -1.68 | Pnkd | --- |
| 1390667_at | -1.68 | Lrtomt | chr1q32 |
| 1369983_at | -1.68 | Ccl5 | chr10q26 |
| 1397165_at | -1.68 | --- | --- |
| 1385497_x_at | -1.68 | Lnx1 | chr14p11 |
| 1390543_at | -1.68 | --- | --- |
| 1368387_at | -1.68 | Bdh1 | chr11q22 |
| 1377673_at | -1.68 | --- | --- |
| 1370336_at | -1.68 | Osgin1 | chr19q12 |
| 1387776_at | -1.69 | Tgm2 | chr3q42 |
| 1370320_at | -1.69 | Pbld | chr20p11 |
| 1388153_at | -1.69 | Acsl1 | chr16q11 |
| 1388743_at | -1.69 | --- | --- |
| 1369654_at | -1.69 | Prkaa2 | chr5q34 |
| 1392534_at | -1.69 | Pmepa1 | chr3q42 |
| 1396213_at | -1.69 | --- | --- |
| 1377877_at | -1.69 | --- | --- |
| 1384542_at | -1.69 | --- | --- |
| 1385585_at | -1.69 | --- | --- |
| 1374187_at | -1.69 | --- | --- |
| 1398520_at | -1.70 | RGD1566112 | chr6q24 |
| 1396407_at | -1.70 | Gas8 | chr19q12 |
| 1391046_at | -1.70 | Smyd3 | chr13q26 |
| 1387925_at | -1.70 | Asns | chr4q13-q21 |
| 1398468_at | -1.70 | --- | --- |
| 1389166_at | -1.70 | Cib2 | chr8q24 |
| 1376119_at | -1.70 | Thada | chr6q12 |
| 1370594_at | -1.70 | Igsf1 | chrXq36 |
| 1389067_at | -1.71 | Slco4a1 | chr3q43 |
| 1374035_at | -1.71 | Rem2 | chr15p13 |
| 1387703_a_at | -1.71 | Usp2 | chr8q22 |
| 1385794_at | -1.72 | MGC94891 | chr1q11 |
| 1376804_at | -1.72 | Myo6 | chr8q31 |
| 1398612_at | -1.72 | Akr1c12 | chr17q12.3 |
| 1381906_at | -1.72 | Tpd52 | chr2q23 |
| 1373963_at | -1.73 | Hdhd3 | chr5q24 |
| 1370628_at | -1.73 | Gzmb | chr15p12 |
| 1380285_at | -1.73 | Chrd | chr11q23 |
| 1376124_at | -1.73 | Iqub | chr4q22 |
| 1368419_at | -1.73 | Cp | chr2q24 |
| 1390578_at | -1.73 | Brsk2 | chr1q41 |
| 1369577_at | -1.73 | Socs2 | chr7q13 |
| 1371542_at | -1.73 | Tuba4a | chr9q33 |
| 1373348_at | -1.73 | --- | --- |
| 1372860_at | -1.73 | Lhpp | chr1q41 |
| 1394641_at | -1.74 | --- | --- |
| 1371566_at | -1.74 | Fbxl22 | chr8q24 |
| 1391209_at | -1.74 | --- | --- |
| 1391547_at | -1.74 | --- | --- |
| 1381924_at | -1.74 | RGD1561507 | chr19q12 |
| 1378082_at | -1.74 | --- | --- |
| 1393933_at | -1.74 | Sorl1 | chr8q22 |
| 1398383_at | -1.74 | Cyb561 | chr10q32.1 |
| 1383164_at | -1.74 | --- | --- |
| 1386689_at | -1.74 | Hook2 | chr19q11 |
| 1393719_at | -1.75 | --- | --- |
| 1381591_at | -1.75 | --- | --- |
| 1393084_at | -1.75 | Hrasls | chr11q22 |
| 1377125_at | -1.75 | Dnajc6 | chr5q33 |
| 1367648_at | -1.75 | Igfbp2 | chr9q33 |
| 1398107_at | -1.75 | Ggct | chr4q24 |
| 1368103_at | -1.76 | Abcg1 | chr20p12 |
| 1394993_at | -1.76 | --- | --- |
| 1373810_at | -1.76 | Pla2g12a | chr2q43 |
| 1380527_at | -1.76 | --- | --- |
| 1380591_at | -1.76 | --- | --- |
| 1381201_at | -1.76 | --- | --- |
| 1369630_at | -1.76 | Adk | chr15p16 |
| 1372182_at | -1.76 | Pfkp | chr17q12.2 |
| 1398533_at | -1.76 | Cyfip2 | chr10q21 |
| 1378856_at | -1.77 | Tceal3 | chrXq35 |
| 1396027_at | -1.77 | --- | --- |
| 1396301_x_at | -1.77 | Afmid | --- |
| 1398016_at | -1.77 | Scd | chr1q54 |
| 1382061_at | -1.77 | Ldhd | chr19q12 |
| 1390507_at | -1.78 | Isg20 | chr1q31 |
| 1377659_at | -1.78 | Mlf1 | chr2q31 |
| 1396771_at | -1.78 | --- | --- |
| 1373159_at | -1.78 | --- | --- |
| 1397462_at | -1.79 | --- | --- |
| 1394458_at | -1.79 | --- | --- |
| 1376336_at | -1.79 | --- | --- |
| 1390987_at | -1.79 | --- | --- |
| 1374942_at | -1.79 | Cpxm2 | chr1q41 |
| 1374677_at | -1.79 | LOC684425 | --- |
| 1389787_at | -1.79 | Ptk7 | chr9q12 |
| 1378245_at | -1.79 | Fam81a | chr8q24 |
| 1375056_at | -1.79 | LOC100361733 | --- |
| 1387138_at | -1.79 | Tac2 | chr7q22 |
| 1381749_at | -1.79 | --- | --- |
| 1396238_at | -1.79 | Galnt14 | chr6q13 |
| 1383993_at | -1.80 | --- | --- |
| 1384420_at | -1.80 | --- | --- |
| 1372665_at | -1.80 | Psat1 | chr1q43 |
| 1387450_at | -1.80 | Tgfa | chr4q34 |
| 1372328_at | -1.80 | Klc4 | chr9q12 |
| 1380403_at | -1.80 | Rwdd2a | chr8q31 |
| 1386891_at | -1.80 | Pebp1 | chr12q16 |
| 1373625_at | -1.81 | Shmt1 | chr10q22 |
| 1382755_at | -1.81 | --- | --- |
| 1376597_at | -1.81 | Zcchc10 | chr10q22 |
| 1387263_at | -1.81 | Pklr | chr2q34 |
| 1393516_at | -1.81 | --- | --- |
| 1368452_at | -1.82 | Abcc6 | chr1q22 |
| 1374943_at | -1.82 | RGD1311378 | chr3q42 |
| 1384846_at | -1.82 | --- | --- |
| 1374081_at | -1.83 | Casc4 | chr3q35 |
| 1375012_at | -1.83 | Pdzd4 | chrXq37 |
| 1381221_at | -1.83 | RGD1311300 | chr15p13 |
| 1373506_at | -1.83 | --- | --- |
| 1393132_at | -1.83 | RGD1560271 | chr9q12 |
| 1378015_at | -1.83 | Ccl21b | chr5q22 |
| 1383798_at | -1.83 | Fam151a | chr5q34 |
| 1378324_at | -1.84 | Ttc30b | chr3q23 |
| 1387946_at | -1.84 | Lgals3bp | chr10q32.3 |
| 1387426_at | -1.84 | Slc25a21 | chr6q23 |
| 1378111_at | -1.84 | --- | --- |
| 1373267_at | -1.84 | Sh3yl1 | chr6q16 |
| 1381253_at | -1.84 | --- | --- |
| 1376710_at | -1.84 | --- | --- |
| 1390790_a_at | -1.85 | --- | --- |
| 1372323_at | -1.85 | Sardh | chr3p12 |
| 1368919_at | -1.85 | Pgf | chr6q31 |
| 1376267_at | -1.85 | --- | --- |
| 1378739_at | -1.85 | Cidec | chr4q42 |
| 1367774_at | -1.85 | Gsta3 | chr9q13 |
| 1383673_at | -1.85 | Nap1l2 | chrXq31 |
| 1390927_at | -1.85 | LOC654482 | chr8q11 |
| 1377821_at | -1.85 | --- | --- |
| 1375905_at | -1.85 | Ttyh1 | chr1q12 |
| 1382145_at | -1.85 | Slc7a4 | chr11q23 |
| 1375493_at | -1.86 | --- | --- |
| 1368887_at | -1.86 | --- | --- |
| 1383148_at | -1.86 | --- | --- |
| 1378471_at | -1.86 | Raver2 | chr5q33 |
| 1398093_at | -1.86 | Nup62cl | chrXq35 |
| 1389652_at | -1.86 | --- | --- |
| 1380123_at | -1.86 | Tmem79 | chr2q34 |
| 1376726_at | -1.87 | --- | --- |
| 1386720_at | -1.87 | Tmprss8 | chr10q12 |
| 1395536_at | -1.87 | --- | --- |
| 1389943_at | -1.87 | --- | --- |
| 1367785_at | -1.87 | Cnn1 | chr8q13 |
| 1382217_at | -1.87 | --- | --- |
| 1393414_at | -1.87 | --- | --- |
| 1387344_at | -1.87 | Aldh6a1 | chr6q31 |
| 1375964_at | -1.87 | Psph | chr12q13 |
| 1369629_at | -1.87 | Adk | chr15p16 |
| 1381476_at | -1.87 | --- | --- |
| 1374417_at | -1.87 | --- | --- |
| 1374273_at | -1.88 | --- | --- |
| 1398348_at | -1.88 | --- | --- |
| 1396325_at | -1.88 | Zfp819 | chr1q22 |
| 1368418_a_at | -1.88 | Cp | chr2q24 |
| 1394735_at | -1.88 | Catsper2 | chr3q35 |
| 1370781_a_at | -1.88 | Kcnip1 | chr10q12 |
| 1390126_at | -1.88 | --- | --- |
| 1382924_at | -1.88 | Pank1 | chr1q52 |
| 1388526_at | -1.88 | Gstz1 | --- |
| 1391880_at | -1.88 | --- | --- |
| 1391292_at | -1.88 | --- | --- |
| 1374556_at | -1.89 | Smox | chr3q36 |
| 1391087_at | -1.89 | Lrrc56 | chr1q41 |
| 1385411_at | -1.90 | Usp43_predicted | chr10q24 |
| 1380132_at | -1.90 | --- | --- |
| 1374995_at | -1.90 | Elmo3 | chr19q11 |
| 1385978_at | -1.90 | --- | --- |
| 1387314_at | -1.90 | Sult1b1 | chr14p21 |
| 1376427_a_at | -1.90 | Gldc | chr1q52 |
| 1375428_at | -1.90 | Creg1 | chr13q23 |
| 1393017_at | -1.90 | Rhpn1 | chr7q34 |
| 1374871_at | -1.90 | Asrgl1 | chr1q43 |
| 1367668_a_at | -1.90 | Scd | chr1q54 |
| 1394581_at | -1.90 | --- | --- |
| 1388051_at | -1.90 | Slc26a3 | chr6q16 |
| 1373148_at | -1.91 | Cpxm2 | chr1q41 |
| 1387908_at | -1.91 | Rasd1 | chr10q22 |
| 1368814_at | -1.91 | Aldh6a1 | chr6q31 |
| 1368674_at | -1.91 | Pygl | chr6q24 |
| 1393440_at | -1.91 | --- | --- |
| 1368990_at | -1.92 | Cyp1b1 | chr6q11 |
| 1398580_at | -1.92 | Wdr31 | chr5q24 |
| 1370638_at | -1.92 | Ank3 | chr20p11 |
| 1380021_at | -1.93 | Dennd2d | chr2q34 |
| 1390366_at | -1.93 | Pcdh24 | chr17p14 |
| 1374634_at | -1.93 | Pih1d2 | chr8q23 |
| 1380388_at | -1.93 | --- | --- |
| 1382010_at | -1.93 | Tmem107 | chr10q24 |
| 1379794_at | -1.94 | Gzmb | chr15p12 |
| 1391018_at | -1.94 | --- | --- |
| 1398430_at | -1.94 | --- | --- |
| 1374762_at | -1.94 | --- | --- |
| 1380596_at | -1.94 | Dsg2 | chr18p12 |
| 1382232_at | -1.94 | --- | --- |
| 1380383_at | -1.95 | Arl4d | chr10q32.1 |
| 1390632_at | -1.95 | --- | --- |
| 1368905_at | -1.95 | Ces2l | chr1q55 |
| 1383838_at | -1.95 | --- | --- |
| 1385924_at | -1.95 | --- | --- |
| 1386870_at | -1.95 | Glul | chr13q22 |
| 1377716_at | -1.96 | --- | --- |
| 1372280_at | -1.96 | Asb2 | chr6q32 |
| 1370367_at | -1.96 | Slc1a1 | chr1q52 |
| 1370148_at | -1.97 | Hp | chr19q12 |
| 1377059_at | -1.97 | --- | --- |
| 1377695_at | -1.97 | Smtnl2 | chr10q24 |
| 1381968_at | -1.97 | Creg1 | chr13q23 |
| 1384304_at | -1.98 | --- | --- |
| 1382983_at | -1.98 | --- | --- |
| 1369977_at | -1.98 | Uchl1 | chr14p11 |
| 1382137_at | -1.99 | Abhd3 | chr18p13 |
| 1367585_a_at | -1.99 | Atp1a1 | chr2q34 |
| 1376226_at | -1.99 | --- | --- |
| 1373790_at | -1.99 | --- | --- |
| 1387104_at | -1.99 | Scnn1a | chr4q42 |
| 1391279_at | -1.99 | Scin | chr6q21 |
| 1376104_at | -1.99 | RGD1308319 | chr9q37 |
| 1390164_at | -1.99 | --- | --- |
| 1379073_at | -2.00 | Slc25a23 | chr9q11 |
| 1367633_at | -2.00 | Glul | chr13q22 |
| 1382356_at | -2.00 | Efhc2 | chrXq12 |
| 1370875_at | -2.00 | Ezr | chr1q11 |
| 1395622_at | -2.01 | --- | --- |
| 1386641_at | -2.01 | --- | --- |
| 1395996_at | -2.01 | --- | --- |
| 1385983_at | -2.01 | --- | --- |
| 1385134_at | -2.02 | T2 | chr1q11 |
| 1383405_at | -2.02 | LOC100364681 | --- |
| 1367892_at | -2.02 | Pdk2 | chr10q31 |
| 1368059_at | -2.03 | Crym | --- |
| 1371121_at | -2.03 | Sert1 | --- |
| 1375733_at | -2.03 | --- | --- |
| 1392982_at | -2.03 | --- | --- |
| 1384479_at | -2.03 | Galnt3 | chr3q21 |
| 1383186_at | -2.03 | RGD1307749 | chr6q32 |
| 1382258_at | -2.03 | Rgnef | chr2q12 |
| 1397827_at | -2.03 | Nanos3 | chr19q11 |
| 1369546_at | -2.04 | Bbox1 | chr3q33 |
| 1385016_at | -2.04 | --- | --- |
| 1379352_at | -2.05 | Pip5k1b | chr1q51 |
| 1390664_at | -2.05 | Tmem116 | chr12q16 |
| 1393825_at | -2.05 | Gna14 | chr1q43 |
| 1377014_at | -2.06 | RGD1308116 | chr2q16 |
| 1381759_at | -2.06 | RGD1565975 | chr19p11 |
| 1368355_at | -2.06 | Myo5b | chr18q12.2-q12.3 |
| 1382932_at | -2.06 | Lrrc50 | chr19q12 |
| 1375997_at | -2.06 | RGD1311756 | chr6q32 |
| 1376877_at | -2.07 | Cdcp1 | chr8q32 |
| 1388287_at | -2.07 | Hoxb7 | chr10q31 |
| 1382384_at | -2.07 | Rassf6 | chr14p22 |
| 1385044_at | -2.07 | Csrnp3 | chr3q21 |
| 1386985_at | -2.07 | Gstm1 | chr2q34 |
| 1390051_at | -2.07 | RGD1562533 | chr12p11 |
| 1397383_at | -2.07 | Emx1 | chr4q34 |
| 1385810_at | -2.07 | Lrtomt | chr1q32 |
| 1373427_at | -2.07 | Rragd | chr5q21 |
| 1373957_at | -2.07 | Reln | chr4q11.2 |
| 1382412_at | -2.08 | --- | --- |
| 1392580_at | -2.08 | --- | --- |
| 1376239_at | -2.08 | Atp6v1c2 | --- |
| 1371363_at | -2.09 | Gpd1 | chr7q36 |
| 1372702_at | -2.09 | Prp2l1 | chr10q32.1 |
| 1376691_at | -2.09 | --- | --- |
| 1370020_at | -2.09 | Slc25a10 | chr10q32.3 |
| 1377051_at | -2.10 | LOC100366119 | --- |
| 1375305_at | -2.10 | Snhg11 | chr3q42 |
| 1393551_at | -2.10 | LOC100365899 | --- |
| 1394786_at | -2.11 | Sorl1 | chr8q22 |
| 1370321_at | -2.11 | Aifm1 | chrXq35 |
| 1381449_s_at | -2.11 | Tgfa | chr4q34 |
| 1384116_at | -2.11 | LOC687208 | --- |
| 1392512_at | -2.11 | Hist3h2ba | chr10q22 |
| 1370217_at | -2.11 | Ddr1 | chr20p12 |
| 1391229_at | -2.11 | Camk1g | chr13q27 |
| 1386568_at | -2.12 | Irf6 | chr13q27 |
| 1383290_at | -2.12 | Spint1 | chr3q35 |
| 1384914_at | -2.12 | --- | --- |
| 1376892_at | -2.13 | Gria3 | chrXq11 |
| 1370228_at | -2.13 | Tf | chr8q32 |
| 1395142_at | -2.13 | --- | --- |
| 1390918_at | -2.13 | Grtp1 | chr16q12.5 |
| 1384440_at | -2.13 | --- | --- |
| 1391944_at | -2.13 | --- | --- |
| 1367999_at | -2.13 | Aldh2 | chr12q16 |
| 1368762_at | -2.14 | Ubd | chr20p12 |
| 1371824_at | -2.14 | Ak3l1 | chr5q33 |
| 1369777_a_at | -2.14 | Shank2 | chr1q41 |
| 1368021_at | -2.14 | Adh1 | chr2q44 |
| 1398646_at | -2.14 | RGD1565493 | chr7q22 |
| 1369921_at | -2.14 | Gstm3 | chr2q34 |
| 1367843_at | -2.15 | Akr7a2 | chr5q36 |
| 1380062_at | -2.15 | Mpp6 | chr4q24 |
| 1396217_at | -2.15 | --- | --- |
| 1395217_at | -2.16 | --- | --- |
| 1385830_at | -2.16 | RGD1561507 | chr19q12 |
| 1397601_at | -2.16 | --- | --- |
| 1392973_at | -2.16 | Nav2 | chr1q22 |
| 1372959_at | -2.16 | Nme4 | chr10q12 |
| 1397180_at | -2.16 | Styxl1 | chr12q12 |
| 1382956_at | -2.17 | LOC361016 | chr15p14 |
| 1380326_at | -2.17 | --- | --- |
| 1397788_at | -2.17 | --- | --- |
| 1388913_at | -2.18 | Ppap2c | chr7q11 |
| 1395384_at | -2.19 | Usp31 | chr1q36 |
| 1382320_at | -2.19 | RGD1307315 | chr6q32 |
| 1392876_at | -2.19 | Rab11fip1 | chr16q12.3-q12.4 |
| 1386454_at | -2.19 | Slc23a3 | chr9q33 |
| 1389306_at | -2.19 | Matn2 | chr7q22 |
| 1394555_at | -2.19 | --- | --- |
| 1389464_at | -2.19 | Lnx1 | chr14p11 |
| 1384302_at | -2.19 | Slc6a17 | --- |
| 1370410_at | -2.19 | Igsf1 | chrXq36 |
| 1382940_at | -2.20 | Glb1l2 | chr8q13 |
| 1390913_at | -2.20 | --- | --- |
| 1387766_a_at | -2.21 | Rbp2 | chr8q31 |
| 1374320_at | -2.21 | F5 | chr13q22 |
| 1368109_at | -2.21 | St3gal5 | chr4q33 |
| 1386914_at | -2.21 | Gmpr | chr17p13 |
| 1393536_at | -2.21 | LOC680590 | chr7q36 |
| 1389274_at | -2.22 | Dcakd | chr10q32.1 |
| 1390172_at | -2.22 | Dhtkd1 | chr17q12.3 |
| 1392556_at | -2.23 | Shroom3 | chr14p22 |
| 1373198_at | -2.23 | Dnajc22 | chr7q36 |
| 1398288_at | -2.24 | Agtr2 | chrXq34 |
| 1380334_at | -2.24 | Rbm47 | chr14p11 |
| 1394829_at | -2.25 | --- | --- |
| 1382581_at | -2.25 | Ehf | chr3q32 |
| 1385491_at | -2.25 | Pnmal2 | chr1q21 |
| 1375230_at | -2.25 | --- | --- |
| 1379530_at | -2.25 | --- | --- |
| 1387808_at | -2.26 | Slc7a7 | chr15p13 |
| 1382630_at | -2.26 | RGD1311558 | chr1q55 |
| 1376313_at | -2.26 | Tpcn2 | chr1q42 |
| 1377142_at | -2.27 | --- | --- |
| 1382084_at | -2.27 | RGD1562988 | chr5q24 |
| 1385717_at | -2.27 | Gdap1 | chr5q11 |
| 1385668_at | -2.27 | LOC683460 | --- |
| 1395020_at | -2.28 | Plekhh1 | chr6q24 |
| 1368245_at | -2.28 | Upb1 | chr20p12 |
| 1367609_at | -2.28 | Mif | chr20p12 |
| 1368501_s_at | -2.28 | Mcpt10 | chr15p12 |
| 1370053_at | -2.29 | Dlgap1 | chr9q38 |
| 1369086_a_at | -2.29 | Cacna1d | chr16p16 |
| 1377048_at | -2.30 | Dak | chr1q43 |
| 1393954_at | -2.30 | Chrdl2 | chr1q32 |
| 1385659_at | -2.30 | LOC682861 | --- |
| 1397678_at | -2.31 | --- | --- |
| 1375011_at | -2.31 | --- | --- |
| 1382712_at | -2.32 | --- | --- |
| 1383286_at | -2.32 | Plek2 | chr6q24 |
| 1372841_at | -2.32 | Reep6 | chr7q11 |
| 1387230_at | -2.33 | Slc12a3 | chr19p14-p12 |
| 1388537_at | -2.33 | Nipsnap1 | chr14q21 |
| 1371776_at | -2.33 | --- | --- |
| 1373057_at | -2.33 | --- | --- |
| 1389114_at | -2.33 | Thnsl2 | chr4q32 |
| 1390454_at | -2.33 | Nipsnap1 | chr14q21 |
| 1395847_at | -2.34 | LOC100188933 | --- |
| 1373036_at | -2.34 | LOC100363987 | --- |
| 1397301_at | -2.35 | --- | --- |
| 1373110_at | -2.35 | --- | --- |
| 1380681_at | -2.37 | Rnf180 | chr2q13 |
| 1373725_at | -2.37 | --- | --- |
| 1374195_at | -2.38 | Lad1 | chr13q13 |
| 1388539_at | -2.38 | Pkp2 | chr11q23 |
| 1394767_at | -2.38 | --- | --- |
| 1368514_at | -2.38 | Maob | chrXq12 |
| 1394027_at | -2.39 | Nup62cl | chrXq35 |
| 1368219_at | -2.39 | Clcn2 | chr11q23 |
| 1374121_at | -2.40 | --- | --- |
| 1372296_at | -2.41 | Sh3bgr | chr11q11 |
| 1390557_at | -2.42 | Gca | chr3q21 |
| 1373580_at | -2.42 | --- | --- |
| 1367908_at | -2.43 | Gcsh | chr19q12 |
| 1368691_at | -2.43 | Gria3 | chrXq11 |
| 1390776_at | -2.43 | Irx3 | chr19p11 |
| 1387932_at | -2.43 | Slc1a1 | chr1q52 |
| 1389362_at | -2.43 | Ptpn3 | chr5q24 |
| 1372895_at | -2.43 | RGD1309676 | chr16p14 |
| 1393338_at | -2.44 | Scx | chr7q34 |
| 1395810_at | -2.44 | --- | --- |
| 1389049_at | -2.44 | --- | --- |
| 1378830_at | -2.44 | --- | --- |
| 1387160_at | -2.44 | Kcne3 | chr1q32 |
| 1379131_at | -2.45 | RGD1562029 | chr9q31 |
| 1394097_at | -2.45 | --- | --- |
| 1392813_at | -2.45 | LOC100361122 | --- |
| 1377790_at | -2.45 | Rab11fip1 | chr16q12.3-q12.4 |
| 1392901_at | -2.45 | Lrrc1 | chr8q31 |
| 1367811_at | -2.45 | Phgdh | chr2q34 |
| 1389777_at | -2.46 | Ribc1 | chrXq21 |
| 1376265_at | -2.46 | Steap2 | chr4q12 |
| 1379697_at | -2.46 | --- | --- |
| 1392279_at | -2.47 | Dnai1 | chr5q22 |
| 1388485_at | -2.47 | Cxcl14 | chr17p14 |
| 1385770_at | -2.47 | Ulk4 | chr8q32 |
| 1394649_at | -2.48 | Ankrd6 | chr5q21 |
| 1393142_at | -2.48 | Cep70 | chr8q31 |
| 1381502_at | -2.48 | Mns1 | chr8q24 |
| 1394622_at | -2.48 | --- | --- |
| 1387804_at | -2.48 | Trim63 | chr5q36 |
| 1374768_at | -2.48 | Ccdc65 | chr7q36 |
| 1368420_at | -2.48 | Cp | chr2q24 |
| 1392809_at | -2.49 | --- | --- |
| 1395327_at | -2.49 | --- | --- |
| 1398250_at | -2.49 | Acot1 | chr6q31 |
| 1383058_at | -2.49 | --- | --- |
| 1388718_at | -2.50 | Tmod1 | chr5q22 |
| 1393075_at | -2.50 | --- | --- |
| 1388926_at | -2.50 | --- | --- |
| 1386960_at | -2.51 | Slc37a4 | chr8q22 |
| 1375775_at | -2.51 | Odf3b | chr7q34 |
| 1381160_at | -2.51 | Tmem200c | chr9q38 |
| 1376728_at | -2.52 | --- | --- |
| 1390530_at | -2.52 | --- | --- |
| 1396129_at | -2.52 | Cxcl14 | chr17p14 |
| 1396614_at | -2.52 | Sfrp2 | chr2q34 |
| 1368465_at | -2.53 | Accn1 | chr10q26 |
| 1381206_at | -2.54 | Plcxd2 | chr11q21 |
| 1383224_at | -2.55 | Pard6b | chr3q42 |
| 1380975_at | -2.55 | --- | --- |
| 1371052_at | -2.56 | Nog | chr10q26 |
| 1368964_at | -2.56 | Lrrn3 | chr6q21 |
| 1370818_at | -2.56 | Decr2 | chr10q12 |
| 1393271_at | -2.57 | Ihh | chr9q33 |
| 1373781_a_at | -2.57 | Sbsn | chr1q21 |
| 1376736_at | -2.57 | --- | --- |
| 1373909_at | -2.57 | --- | --- |
| 1376546_at | -2.57 | RGD1565432 | chr1q22 |
| 1368367_at | -2.57 | Cuzd1 | chr1q41 |
| 1385974_at | -2.58 | Pard6g | chr18q12.3 |
| 1386538_at | -2.58 | RGD1310495 | chr20q12 |
| 1383897_at | -2.58 | H2afy2 | chr20q11 |
| 1385714_at | -2.58 | Tmc4 | chr1q12 |
| 1373216_at | -2.58 | LOC691849 | chr8q24 |
| 1373977_at | -2.59 | Kif5c | chr3q12 |
| 1372654_at | -2.59 | Eps8l2 | chr1q41 |
| 1392909_at | -2.59 | --- | --- |
| 1368283_at | -2.59 | Ehhadh | chr11q23 |
| 1378273_at | -2.59 | --- | --- |
| 1386931_at | -2.60 | Tnni3 | chr1q12 |
| 1387370_at | -2.60 | Tmod1 | chr5q22 |
| 1383188_at | -2.60 | --- | --- |
| 1369439_at | -2.61 | Agtr1b | chr2q24 |
| 1380828_at | -2.62 | Gabra1 | chr10q21 |
| 1372920_at | -2.62 | Prodh | chr11q23 |
| 1368260_at | -2.62 | Aurkb | chr10q24 |
| 1385038_at | -2.63 | Hhip | chr19q11 |
| 1368016_at | -2.63 | Pecr | chr9q33 |
| 1368528_at | -2.63 | Cd99l2 | chr15p16 |
| 1394866_at | -2.64 | --- | --- |
| 1393335_at | -2.64 | Egfl6 | chrXq21 |
| 1370453_at | -2.65 | Tex101 | chr1q21 |
| 1379144_at | -2.65 | Rab36 | chr20p12 |
| 1398653_at | -2.65 | LOC683514 | --- |
| 1398398_at | -2.65 | Hoxa10 | chr4q24 |
| 1378163_at | -2.65 | Grem2 | chr13q24 |
| 1385994_at | -2.66 | Spo11 | chr3q42 |
| 1388605_at | -2.67 | --- | --- |
| 1392545_at | -2.67 | Tcfap2b | chr9q13 |
| 1379031_at | -2.67 | Gca | chr3q21 |
| 1387802_at | -2.67 | Dlgap1 | chr9q38 |
| 1391976_at | -2.67 | Six4 | chr6q24 |
| 1391936_a_at | -2.68 | --- | --- |
| 1385000_at | -2.68 | RGD1565611 | chr10q24 |
| 1376310_at | -2.70 | --- | --- |
| 1386647_at | -2.70 | Map7 | chr1p12 |
| 1387793_at | -2.70 | Slc9a3r1 | chr10q32.2 |
| 1391492_at | -2.71 | --- | --- |
| 1378090_at | -2.71 | --- | --- |
| 1384431_at | -2.71 | --- | --- |
| 1389054_at | -2.71 | LOC498368 | chr14p11 |
| 1375495_at | -2.72 | --- | --- |
| 1367853_at | -2.72 | Slc12a2 | chr18q12.1 |
| 1398343_at | -2.74 | Dnaja4 | chr8q24 |
| 1372755_at | -2.74 | Mal2 | chr7q31 |
| 1368627_at | -2.74 | Rgn | chrXq11-q12 |
| 1376945_at | -2.74 | --- | --- |
| 1379293_at | -2.75 | Gzma | chr2q14 |
| 1384277_at | -2.75 | Plekhh1 | chr6q24 |
| 1376203_at | -2.76 | --- | --- |
| 1378681_at | -2.76 | --- | --- |
| 1378002_at | -2.77 | Hspa4l | chr2q26 |
| 1375026_at | -2.77 | Calml4 | chr8q24 |
| 1369050_at | -2.78 | Pik3c2g | chr4q44 |
| 1397736_at | -2.79 | Cldn22 | chr16q11 |
| 1387178_a_at | -2.79 | Cbs | chr20p12 |
| 1382698_at | -2.80 | Sim1 | chr20q13 |
| 1397766_at | -2.82 | --- | --- |
| 1390518_at | -2.83 | Emid1 | chr14q21 |
| 1389678_at | -2.83 | --- | --- |
| 1382291_at | -2.83 | --- | --- |
| 1390119_at | -2.83 | Sfrp2 | chr2q34 |
| 1384197_at | -2.85 | Dlgap1 | chr9q38 |
| 1383991_at | -2.86 | Lrrc8e | chr12p12 |
| 1377013_at | -2.87 | --- | --- |
| 1382612_at | -2.87 | --- | --- |
| 1383165_at | -2.88 | RGD1310209 | chr2q34 |
| 1388916_at | -2.89 | --- | --- |
| 1374859_at | -2.90 | --- | --- |
| 1379374_at | -2.90 | Lppr4 | chr2q41 |
| 1384276_at | -2.90 | --- | --- |
| 1389223_at | -2.90 | --- | --- |
| 1392852_at | -2.90 | --- | --- |
| 1391840_at | -2.91 | Rsph9 | chr9q12 |
| 1397010_at | -2.93 | --- | --- |
| 1368237_at | -2.94 | Tnmd | chrXq34 |
| 1369412_a_at | -2.96 | Slc19a1 | chr20p12 |
| 1370334_at | -2.96 | Plekhb1 | chr1q32 |
| 1387483_at | -2.97 | Plcg2 | chr19q12 |
| 1381557_at | -2.97 | Gna14 | chr1q43 |
| 1388102_at | -2.98 | Ptgr1 | chr5q24 |
| 1392012_at | -2.99 | LOC686567 | --- |
| 1373006_at | -3.00 | Tmem171 | chr2q12 |
| 1376776_at | -3.00 | Ccdc17 | chr5q36 |
| 1395518_at | -3.01 | --- | --- |
| 1370813_at | -3.01 | Gstm5 | chr2q34 |
| 1392754_at | -3.02 | Adam8 | chr1q41 |
| 1396300_at | -3.03 | Afmid | --- |
| 1387034_at | -3.03 | Pah | chr7q13 |
| 1393841_at | -3.04 | RGD1310773 | chr5q22 |
| 1390371_at | -3.05 | LOC100359739 | --- |
| 1378470_at | -3.07 | Paqr5 | chr8q24 |
| 1383554_at | -3.07 | Rnf128 | chrXq35 |
| 1385251_at | -3.08 | Fam110c | chr6q16 |
| 1389622_at | -3.08 | Slc25a13 | chr4q13 |
| 1389783_s_at | -3.08 | Fcgbp | chr1q21 |
| 1367682_at | -3.09 | Mdk | chr3q24 |
| 1391352_at | -3.10 | Erp27 | chr4q43 |
| 1384863_at | -3.11 | Cpne7 | chr19q12 |
| 1377032_at | -3.11 | --- | --- |
| 1378753_at | -3.12 | Ocln | chr2q12 |
| 1380318_at | -3.12 | --- | --- |
| 1376657_at | -3.13 | Cadm1 | chr8q23 |
| 1386998_at | -3.14 | Aldoc | chr10q31-q32.1 |
| 1380929_at | -3.15 | RGD1560244 | chr10q12 |
| 1372613_at | -3.15 | Bdh2 | chr2q43 |
| 1394928_at | -3.16 | Xkr4 | chr5q12 |
| 1374070_at | -3.18 | Gpx2 | chr6q24 |
| 1389579_at | -3.18 | --- | --- |
| 1395625_at | -3.21 | --- | --- |
| 1379252_at | -3.22 | --- | --- |
| 1382835_at | -3.24 | --- | --- |
| 1380120_at | -3.24 | --- | --- |
| 1391675_at | -3.25 | RGD1309708 | chr13q22 |
| 1393064_at | -3.25 | --- | --- |
| 1380424_at | -3.26 | Ubxn11 | chr5q36 |
| 1384028_at | -3.27 | --- | --- |
| 1379135_at | -3.27 | Ttll3 | chr4q42 |
| 1367909_at | -3.28 | Dcxr | --- |
| 1387990_at | -3.28 | Nrg2 | chr18p11 |
| 1374742_at | -3.28 | --- | --- |
| 1387831_at | -3.29 | Xcl1 | chr13q23 |
| 1367858_at | -3.30 | Mmp11 | chr20p12 |
| 1379656_a_at | -3.30 | --- | --- |
| 1387949_at | -3.31 | Cyp2c22 | chr1q53 |
| 1383497_at | -3.31 | --- | --- |
| 1383486_at | -3.31 | --- | --- |
| 1372372_at | -3.32 | Cmbl | chr2q22 |
| 1377375_at | -3.32 | Aass | chr4q22 |
| 1390809_at | -3.32 | --- | --- |
| 1369581_at | -3.34 | Pemt | chr10q22 |
| 1371492_at | -3.35 | Apobec2 | chr9q12 |
| 1380393_at | -3.36 | Cryz | chr2q45 |
| 1369877_at | -3.38 | Cd8a | chr4q33 |
| 1372103_at | -3.39 | Dnaja4 | chr8q24 |
| 1396297_at | -3.39 | --- | --- |
| 1395072_at | -3.40 | --- | --- |
| 1388274_at | -3.40 | Bmyc | chr3p13 |
| 1387491_at | -3.42 | Gk | chrXq22 |
| 1387730_at | -3.42 | Pax8 | chr3p13 |
| 1368378_at | -3.44 | Aldh1l1 | --- |
| 1388545_at | -3.45 | --- | --- |
| 1368350_at | -3.46 | Ptprz1 | chr4q22 |
| 1370517_at | -3.47 | Nptx1 | chr10q32.3 |
| 1374963_s_at | -3.48 | --- | --- |
| 1387011_at | -3.50 | Lcn2 | chr3p11 |
| 1387313_at | -3.51 | Myoc | chr13q22 |
| 1385622_at | -3.51 | --- | --- |
| 1371496_at | -3.54 | LOC100366121 | --- |
| 1368188_at | -3.55 | Hpd | chr12q16 |
| 1389649_at | -3.56 | --- | --- |
| 1393246_at | -3.57 | Zmynd10 | chr8q32 |
| 1382874_at | -3.58 | Npl | chr13q21 |
| 1393098_at | -3.58 | Llgl2 | chr10q32.3 |
| 1379229_at | -3.61 | --- | --- |
| 1368191_a_at | -3.62 | Slc22a1 | chr1q11-q12 |
| 1387477_at | -3.63 | Kcnk12 | chr6q12 |
| 1378348_at | -3.64 | Tmem200c | chr9q38 |
| 1383496_at | -3.64 | RGD1560481 | chr5q13 |
| 1392694_at | -3.64 | RGD1562344 | chr2q34 |
| 1388780_at | -3.65 | Terf2ip | chr19q12 |
| 1372170_at | -3.65 | Acy1 | chr8q32 |
| 1374251_at | -3.68 | --- | --- |
| 1381352_at | -3.69 | Wdr38 | chr3q11 |
| 1371677_at | -3.70 | Spink8 | chr8q32 |
| 1386774_at | -3.71 | Fam5b | chr13q22 |
| 1396103_at | -3.72 | Cmbl | chr2q22 |
| 1384868_at | -3.73 | --- | --- |
| 1389662_at | -3.73 | Wnk4 | chr10q32.1 |
| 1388802_at | -3.74 | Bex1 | chrXq35 |
| 1381554_at | -3.74 | --- | --- |
| 1379741_at | -3.74 | Atp6v0a4 | chr4q22 |
| 1380567_at | -3.74 | --- | --- |
| 1375575_at | -3.75 | Camk2b | chr14q21 |
| 1388320_at | -3.76 | Spint2 | chr1q21 |
| 1374427_at | -3.77 | --- | --- |
| 1391459_at | -3.77 | --- | --- |
| 1375934_at | -3.79 | --- | --- |
| 1385414_at | -3.79 | Cd8a | chr4q33 |
| 1383853_at | -3.81 | Dyrk3 | chr13q13 |
| 1389735_at | -3.81 | Rps6ka6 | chrXq31 |
| 1378930_a_at | -3.81 | --- | --- |
| 1389801_at | -3.81 | Fgfr4 | chr17p14 |
| 1387220_at | -3.82 | Mcpt9 | chr15p12 |
| 1385614_at | -3.86 | Mlph | chr9q36 |
| 1377351_at | -3.86 | Susd3 | chr17p14 |
| 1374628_at | -3.86 | Cryz | chr2q45 |
| 1382458_at | -3.86 | --- | --- |
| 1393935_at | -3.89 | Tmem139 | chr4q23 |
| 1367949_at | -3.90 | Penk | chr5q12 |
| 1377311_at | -3.91 | Emx2 | chr1q55 |
| 1368209_at | -3.91 | Pdzk1ip1 | chr5q36 |
| 1374622_at | -3.93 | --- | --- |
| 1373315_at | -3.95 | Arnt2 | chr1q31 |
| 1372277_at | -3.98 | LOC641316 | chr5q36 |
| 1372087_at | -3.99 | Iah1 | chr6q16 |
| 1379960_at | -4.01 | --- | --- |
| 1373991_at | -4.01 | --- | --- |
| 1379458_at | -4.03 | Klhl14 | chr18p12 |
| 1385629_at | -4.03 | RGD1306233 | chr3p12 |
| 1383221_at | -4.04 | RGD1563547 | chr10q12 |
| 1378021_at | -4.06 | --- | --- |
| 1390917_at | -4.06 | --- | --- |
| 1374657_at | -4.14 | Anks4b | --- |
| 1378794_at | -4.15 | --- | --- |
| 1377492_at | -4.24 | Rprml | chr10q32.1 |
| 1380142_at | -4.25 | Hoxb8 | chr10q31 |
| 1388176_at | -4.27 | Cml5 | chr4q34 |
| 1391749_a_at | -4.28 | Nxnl2 | chr17p14 |
| 1370352_at | -4.29 | Cesl1 | --- |
| 1369286_at | -4.31 | Proc | chr18p12 |
| 1370342_at | -4.31 | Kcnk2 | chr13q26 |
| 1387073_at | -4.32 | Snap25 | chr3q36 |
| 1373798_at | -4.33 | RGD1309139 | chr2q34 |
| 1393553_at | -4.34 | --- | --- |
| 1388172_at | -4.38 | Slc22a25 | chr1q43 |
| 1377046_at | -4.46 | Ankrd6 | chr5q21 |
| 1377974_at | -4.51 | --- | --- |
| 1375083_at | -4.51 | --- | --- |
| 1376702_at | -4.51 | Mlc1 | chr7q34 |
| 1391629_at | -4.56 | --- | --- |
| 1375062_at | -4.56 | RGD1561490 | chr8q13 |
| 1372297_at | -4.58 | Gsta4 | chr8q31 |
| 1381774_at | -4.61 | --- | --- |
| 1383458_at | -4.63 | --- | --- |
| 1378800_at | -4.63 | --- | --- |
| 1374488_at | -4.67 | Gramd1b | chr8q22 |
| 1383719_at | -4.67 | Map7 | chr1p12 |
| 1379150_at | -4.71 | --- | --- |
| 1378864_at | -4.72 | Lrrc23 | chr4q42 |
| 1377508_at | -4.72 | --- | --- |
| 1387179_at | -4.76 | Adcy8 | chr7q33 |
| 1378972_at | -4.76 | Wwc1 | chr10q12 |
| 1395372_at | -4.77 | --- | --- |
| 1391656_at | -4.77 | Cpm | chr7q22 |
| 1381819_at | -4.82 | --- | --- |
| 1391323_at | -4.82 | Tf | chr8q32 |
| 1370821_at | -4.83 | Tpmt | chr17p13 |
| 1387375_at | -4.87 | Khk | chr6q14 |
| 1373458_at | -4.90 | Bex4 | chrXq35 |
| 1390863_at | -4.92 | Slc19a2 | chr13q22 |
| 1369705_at | -4.93 | Slc6a20 | chr8q32 |
| 1374122_at | -4.95 | --- | --- |
| 1383575_at | -4.95 | Ctnnd2 | chr2q22 |
| 1389438_at | -4.98 | RGD1561916 | chr5q22 |
| 1380523_at | -5.03 | Fbxo15 | chr18q12.3 |
| 1386937_at | -5.03 | Atp1b1 | chr13q23 |
| 1376074_at | -5.05 | Rab11fip4 | chr10q25 |
| 1373478_at | -5.05 | --- | --- |
| 1390146_at | -5.06 | Tmem150c | chr14p22 |
| 1381976_at | -5.09 | --- | --- |
| 1388506_at | -5.13 | Dsp | chr17p12 |
| 1391661_at | -5.13 | Gk | chrXq22 |
| 1391765_at | -5.14 | Lrrc48 | chr10q22 |
| 1381780_at | -5.14 | --- | --- |
| 1383757_at | -5.15 | --- | --- |
| 1370088_at | -5.15 | Spa17 | chr8q21 |
| 1371922_at | -5.18 | Car12 | chr8q24 |
| 1377033_at | -5.20 | Serpinf2 | chr10q24 |
| 1393706_at | -5.24 | Steap1 | chr4q12 |
| 1374939_at | -5.25 | Cyfip2 | chr10q21 |
| 1389716_at | -5.26 | LOC691918 | chr3q35 |
| 1376327_at | -5.27 | Tnfrsf14 | chr5q36 |
| 1385722_at | -5.31 | Sim2 | chr11q11 |
| 1397919_at | -5.31 | Ccdc37 | chr4q34 |
| 1388433_at | -5.33 | Krt19 | chr10q32.1 |
| 1387336_at | -5.33 | Nat8 | chr4q34 |
| 1374142_at | -5.33 | Prr15 | chr4q24 |
| 1378536_at | -5.38 | Hook1 | chr5q33 |
| 1382111_at | -5.39 | Glod5 | chrXq13 |
| 1378531_at | -5.41 | --- | --- |
| 1377379_at | -5.42 | Irf6 | chr13q27 |
| 1370824_at | -5.44 | Slc38a3 | chr8q32 |
| 1385263_at | -5.48 | Myo7b | chr18p12 |
| 1375936_at | -5.50 | Dsc2 | chr18p12 |
| 1391051_at | -5.51 | --- | --- |
| 1367814_at | -5.57 | Atp1b1 | chr13q23 |
| 1387973_at | -5.58 | Cyp4f4 | chr7q11 |
| 1381603_at | -5.58 | LOC502684 | chr3q41 |
| 1381922_at | -5.59 | Slc5a11 | chr1q36 |
| 1372158_at | -5.65 | Macrod1 | chr1q43 |
| 1387868_at | -5.67 | Lbp | chr3q42 |
| 1398264_at | -5.71 | Slc30a2 | chr5q36 |
| 1369500_at | -5.71 | Kcnk1 | chr19q12 |
| 1391878_at | -5.75 | RGD1560020_predicted | chr1p12 |
| 1389193_at | -5.76 | Sorcs2 | chr14q21 |
| 1398216_at | -5.78 | --- | --- |
| 1379833_at | -5.84 | Lingo4 | chr2q34 |
| 1391542_at | -5.84 | --- | --- |
| 1369335_at | -5.88 | Spef2 | chr2q16 |
| 1394881_at | -5.90 | Fam81a | chr8q24 |
| 1373218_at | -5.94 | --- | --- |
| 1383851_at | -5.95 | Ccdc96 | chr14q21 |
| 1368248_at | -5.97 | Cds1 | chr14p22 |
| 1376561_at | -5.98 | Fbxo16 | chr15p12 |
| 1384831_at | -6.00 | Slc7a13 | chr5q13 |
| 1368060_at | -6.01 | Hrsp12 | chr7q22 |
| 1385602_at | -6.04 | RGD1306233 | chr3p12 |
| 1382918_at | -6.10 | LOC685001 | chr4q11 |
| 1383385_at | -6.11 | --- | --- |
| 1388547_at | -6.14 | Cldn4 | chr12q12 |
| 1393627_at | -6.15 | Osap | chr2q26 |
| 1381927_at | -6.16 | LOC100365876 | --- |
| 1374022_at | -6.17 | --- | --- |
| 1376765_at | -6.21 | Mro | chr18q12.2 |
| 1383395_at | -6.25 | Agmat | chr5q36 |
| 1387088_at | -6.30 | Gal | chr1q42 |
| 1393069_at | -6.33 | Sfrp5 | chr1q54 |
| 1373902_at | -6.36 | Rimklb | chr4q42 |
| 1380342_at | -6.38 | LOC680885 | chr16p16 |
| 1393473_at | -6.39 | RGD1563866 | chr8q24 |
| 1388103_at | -6.42 | Tmem37 | --- |
| 1381800_at | -6.46 | --- | --- |
| 1374699_at | -6.50 | Fam84a | chr6q15 |
| 1389770_at | -6.59 | Ttll9 | chr3q41 |
| 1386823_at | -6.59 | Irx5 | chr19p11 |
| 1381901_at | -6.61 | Tmem212 | chr2q24 |
| 1387924_at | -6.61 | Ngef | chr9q35 |
| 1368344_at | -6.64 | Gad1 | chr3q21 |
| 1393337_at | -6.72 | Tcfcp2l1 | chr13q11 |
| 1382967_at | -6.74 | Gpr64 | chrXq21 |
| 1378674_at | -6.82 | En2 | chr4q11 |
| 1380261_at | -7.04 | LOC499806 | chr3q21 |
| 1384830_at | -7.04 | Dnali1 | chr5q36 |
| 1385049_at | -7.15 | --- | --- |
| 1386571_at | -7.23 | RGD1562626 | chr10q26 |
| 1381776_at | -7.27 | Tmed6 | chr19q12 |
| 1390672_at | -7.28 | Rprm | chr3q12 |
| 1393715_s_at | -7.41 | T2 | chr1q11 |
| 1372485_at | -7.44 | Pcbd1 | chr20q11 |
| 1379566_at | -7.44 | Rbm11 | chr11p11 |
| 1380133_at | -7.45 | Osr2 | chr7q22 |
| 1391457_a_at | -7.45 | Irx2 | chr17p14 |
| 1392916_at | -7.47 | Map7 | chr1p12 |
| 1391293_at | -7.49 | --- | --- |
| 1387434_at | -7.53 | Slc22a4 | --- |
| 1368121_at | -7.54 | Akr7a3 | chr5q36 |
| 1379027_at | -7.55 | Wwc1 | chr10q12 |
| 1383943_at | -7.57 | Dnah7 | chr9q22 |
| 1398625_at | -7.69 | --- | --- |
| 1386691_at | -7.70 | Fbxo36 | chr9q35 |
| 1375856_at | -7.71 | --- | --- |
| 1397896_at | -7.73 | RGD1566400 | chr13q12 |
| 1396229_at | -7.74 | --- | --- |
| 1391206_at | -7.79 | RGD1311892 | chr13q13 |
| 1374512_at | -7.79 | LOC684314 | --- |
| 1389488_at | -7.83 | --- | --- |
| 1389806_at | -7.86 | --- | --- |
| 1391853_at | -7.95 | --- | --- |
| 1368733_at | -7.97 | Sult1e1 | chr14p21.3-p21.2 |
| 1369799_at | -8.00 | Abat | chr10q12 |
| 1382433_at | -8.01 | --- | --- |
| 1398713_at | -8.02 | --- | --- |
| 1379076_at | -8.06 | --- | --- |
| 1379587_at | -8.08 | Rdh5 | chr7q11 |
| 1390421_at | -8.10 | --- | --- |
| 1373436_at | -8.13 | RGD1306739 | chr20p11 |
| 1385577_at | -8.19 | --- | --- |
| 1378374_at | -8.31 | RGD1560137 | chr15q11 |
| 1397500_x_at | -8.34 | --- | --- |
| 1393952_at | -8.47 | Ccdc68 | chr18q12.1 |
| 1378292_at | -8.58 | --- | --- |
| 1368338_at | -8.61 | Cd52 | chr5q36 |
| 1393893_at | -8.62 | Morn5 | chr3p11 |
| 1371530_at | -8.65 | Krt8 | chr7q36 |
| 1391647_at | -8.69 | --- | --- |
| 1394517_at | -8.75 | --- | --- |
| 1384874_at | -8.78 | LOC685001 | chr4q11 |
| 1396486_x_at | -8.81 | RGD1564162 | chr20p12 |
| 1368205_at | -8.88 | Cfi | chr2q43 |
| 1389759_at | -8.90 | Celsr1 | chr7q34 |
| 1390191_at | -9.06 | --- | --- |
| 1396390_at | -9.27 | LOC100270669 | --- |
| 1367838_at | -9.44 | Cth | chr2q45 |
| 1384275_at | -9.44 | LOC688328 | --- |
| 1387877_at | -9.47 | Ftcd | chr20p12 |
| 1381039_at | -9.50 | Dnah1 | chr16p16 |
| 1376711_at | -9.72 | Cldn11 | chr2q24 |
| 1386705_at | -9.78 | --- | --- |
| 1372626_at | -9.85 | Tpd52l1 | chr1p11 |
| 1387240_at | -9.86 | Rdh7 | chr7q22 |
| 1368374_a_at | -9.91 | Ggt1 | --- |
| 1392182_at | -9.95 | --- | --- |
| 1385014_at | -9.96 | Nudt11 | chrXq13 |
| 1367592_at | -9.96 | Tnnt2 | chr13q13 |
| 1385850_at | -9.97 | --- | --- |
| 1384230_at | -9.98 | LOC683980 | --- |
| 1398185_at | -10.03 | Mfap3l | chr16p12 |
| 1393743_at | -10.08 | --- | --- |
| 1367871_at | -10.10 | Cyp2e1 | chr1q41-q42 |
| 1393785_at | -10.15 | RGD1305311 | chr9q35 |
| 1370138_at | -10.17 | Lef1 | chr2q34-q45 |
| 1368115_at | -10.27 | Cldn3 | --- |
| 1398458_at | -10.30 | Wnk2 | chr17p14 |
| 1389648_at | -10.30 | Ripk4 | chr11q12 |
| 1383303_at | -10.33 | Acsm3 | chr1q35 |
| 1384027_a_at | -10.35 | --- | --- |
| 1371089_at | -10.36 | Gsta5 | chr9q13 |
| 1368740_at | -10.37 | P2rx6 | chr11q23 |
| 1374984_at | -10.41 | Epb4.1l5 | chr13q11 |
| 1384789_at | -10.42 | Tekt4 | chr10q12 |
| 1373386_at | -10.62 | Gjb2 | chr15p12 |
| 1387022_at | -10.67 | Aldh1a1 | chr1q51 |
| 1389654_at | -10.73 | Pls1 | chr8q31 |
| 1374910_at | -10.75 | Celsr2 | chr2q34 |
| 1377721_at | -10.76 | Pacrg | chr1q11 |
| 1371099_at | -10.77 | --- | --- |
| 1373329_at | -10.79 | Tmprss2 | chr11q12 |
| 1376501_at | -10.82 | Arhgap8 | chr7q34 |
| 1370696_at | -10.84 | --- | --- |
| 1387023_at | -10.89 | Gstm7 | chr2q34 |
| 1370076_at | -11.14 | Kcnj16 | chr10q32.1 |
| 1368377_at | -11.22 | Gzmc | chr15p12 |
| 1370009_at | -11.23 | --- | --- |
| 1368278_at | -11.27 | Lgals2 | chr7q34 |
| 1391534_at | -11.43 | Elovl2 | chr17p12 |
| 1387037_at | -11.65 | Cubn | chr17q12.3 |
| 1385591_at | -11.70 | --- | --- |
| 1368064_a_at | -11.84 | Ddc | chr14q21 |
| 1395112_at | -11.94 | --- | --- |
| 1368608_at | -12.15 | Cyp2f4 | chr1q21 |
| 1383557_at | -12.20 | --- | --- |
| 1397510_at | -12.27 | --- | --- |
| 1370894_at | -12.31 | Cldn7 | chr10q24 |
| 1387091_at | -12.57 | Padi2 | chr5q36 |
| 1382474_at | -12.58 | Col13a1 | chr20q11 |
| 1376733_at | -12.60 | Igsf11 | chr11q21 |
| 1393206_at | -12.65 | --- | --- |
| 1372208_at | -12.88 | Ppp1r1b | chr10q31 |
| 1382147_at | -12.98 | Tmem132c | chr12q14 |
| 1372423_at | -12.98 | Perp | chr1p12 |
| 1378710_at | -13.20 | Ccdc67 | chr8q12 |
| 1368431_at | -13.23 | Hpn | chr1q21 |
| 1393845_a_at | -13.24 | Tmc4 | chr1q12 |
| 1378567_at | -13.38 | Tjp3 | chr7q11 |
| 1377666_at | -13.43 | Chdh | chr16p16 |
| 1380669_at | -13.52 | --- | --- |
| 1382083_at | -13.53 | Coch | chr6q22 |
| 1394730_at | -13.55 | Gstm6l | chr2q34 |
| 1367917_at | -13.66 | Cyp2d2 | chr7q34 |
| 1389177_at | -13.71 | Perp | chr1p12 |
| 1383879_at | -13.72 | LOC688163 | --- |
| 1376248_at | -13.86 | Sult2b1 | chr1q22 |
| 1384743_at | -13.87 | --- | --- |
| 1379393_at | -13.89 | Vil1 | chr9q33 |
| 1382439_at | -13.90 | Itgb6 | chr3q21 |
| 1382954_at | -13.91 | --- | --- |
| 1388155_at | -14.07 | Krt18 | chr7q36 |
| 1374119_at | -14.27 | Elf3 | chr13q13 |
| 1391520_at | -14.68 | --- | --- |
| 1383425_at | -14.71 | --- | --- |
| 1392099_at | -14.95 | --- | --- |
| 1390765_at | -15.09 | Wfdc2 | chr3q42 |
| 1368782_at | -15.31 | Sstr2 | chr10q32.1 |
| 1395381_at | -15.39 | --- | --- |
| 1375032_at | -15.45 | --- | --- |
| 1384145_at | -15.45 | --- | --- |
| 1385393_at | -15.89 | --- | --- |
| 1383648_x_at | -16.06 | Crb3 | chr9q11 |
| 1381531_at | -16.10 | --- | --- |
| 1392841_at | -16.13 | --- | --- |
| 1387013_at | -16.43 | Tmem27 | chrXq21 |
| 1369928_at | -16.50 | Acta1 | chr19q12 |
| 1384529_at | -16.51 | --- | --- |
| 1388948_at | -16.62 | Stard10 | chr1q32 |
| 1370436_at | -16.69 | Acsm2 | chr1q35 |
| 1394571_at | -16.70 | RGD1305939 | chr7q34 |
| 1368440_at | -16.72 | Slc3a1 | chr6q12 |
| 1368467_at | -16.87 | Cyp4f1 | chr7q11 |
| 1368727_at | -16.91 | Slc7a9 | chr1q21 |
| 1390413_at | -16.96 | RGD1310371 | chr1q31 |
| 1391409_at | -17.06 | Amn | chr6q32 |
| 1384312_at | -17.32 | Irx1 | chr17p14 |
| 1395924_at | -17.36 | --- | --- |
| 1385821_at | -17.36 | --- | --- |
| 1387123_at | -17.75 | Cyp17a1 | chr1q55 |
| 1391539_at | -17.78 | --- | --- |
| 1386943_at | -17.81 | Pllp | chr19p12 |
| 1383437_at | -18.12 | Rab17 | chr9q36 |
| 1372980_at | -18.13 | Tspan33 | chr4q22 |
| 1378042_at | -18.18 | Tmem212 | chr2q24 |
| 1380597_at | -18.23 | --- | --- |
| 1393297_at | -18.29 | Pou2af1 | chr8q24 |
| 1375933_at | -18.56 | Cldn2 | chrXq35 |
| 1377803_at | -18.71 | Ccdc153 | chr8q22 |
| 1387671_at | -19.00 | Sctr | --- |
| 1398326_at | -19.04 | Chchd10 | chr20p12 |
| 1368622_at | -19.55 | Fbp2 | chr17p14 |
| 1390532_at | -19.56 | --- | --- |
| 1376328_at | -19.60 | RGD1310819 | chr9q21 |
| 1380270_at | -19.61 | Mfi2 | chr11q22 |
| 1391228_at | -19.62 | Rsph1 | chr20p12 |
| 1385436_at | -19.65 | Tmc4 | chr1q12 |
| 1384917_at | -19.65 | Ccno | chr2q14 |
| 1382218_at | -19.89 | RGD1305807 | chr5q22 |
| 1380218_at | -19.91 | Cdh29 | chr8q32 |
| 1393263_at | -20.24 | --- | --- |
| 1398621_at | -20.24 | Ak7 | chr6q32 |
| 1392948_at | -20.37 | --- | --- |
| 1393319_a_at | -20.58 | Rab17 | chr9q36 |
| 1387889_at | -21.04 | Folr1 | chr1q32 |
| 1384438_at | -21.30 | --- | --- |
| 1367923_at | -21.41 | Acsbg1 | chr8q22-q24 |
| 1367692_at | -21.52 | Sbp | chr10q12 |
| 1375707_at | -21.53 | --- | --- |
| 1383956_at | -21.63 | RGD1565709 | chr4q42 |
| 1393732_at | -21.70 | --- | --- |
| 1383293_at | -21.80 | --- | --- |
| 1384960_at | -21.81 | Cftr | chr4q21 |
| 1378692_at | -21.87 | Pax8 | chr3p13 |
| 1368672_at | -22.03 | Arg2 | chr6q24 |
| 1370372_at | -22.60 | Rasd2 | chr19p11 |
| 1370379_at | -22.61 | Prss8 | chr1q36 |
| 1393386_at | -22.62 | Fam183b | chr10q22 |
| 1389799_at | -22.67 | --- | --- |
| 1383647_a_at | -22.67 | Crb3 | chr9q11 |
| 1395456_at | -22.92 | --- | --- |
| 1393039_a_at | -23.23 | Rab17 | chr9q36 |
| 1387972_at | -23.64 | Mucdhl | chr1q41 |
| 1368150_at | -23.69 | Slc27a2 | chr3q36 |
| 1374478_at | -23.70 | RGD1305347 | chr5q36 |
| 1367952_at | -23.81 | Lrp2 | chr3q21 |
| 1382467_at | -24.02 | Bex1 /// Bex2 | chrXq35 |
| 1368250_at | -24.58 | Tekt1 | chr10q24 |
| 1384885_at | -24.76 | Tekt2 | chr5q36 |
| 1378340_at | -25.06 | LOC100363796 | --- |
| 1376163_at | -25.07 | Clrn3 | chr1q41 |
| 1381340_at | -25.31 | Rsph10b | chr12p11 |
| 1382751_at | -26.37 | Wfdc10 | chr3q42 |
| 1368160_at | -26.69 | Igfbp1 | chr14q21 |
| 1385383_at | -26.88 | Ccdc113 | chr19p13 |
| 1383895_at | -27.30 | Dynlrb2 | chr19q12 |
| 1368785_a_at | -27.90 | Pitx2 | chr2q42 |
| 1390822_at | -28.24 | Cdh16 | chr19p14 |
| 1378896_at | -28.58 | Slc30a2 | chr5q36 |
| 1373330_at | -28.65 | RGD1560214 | chr10q32.3 |
| 1379513_at | -28.95 | Tmem30b | chr6q24 |
| 1385656_at | -29.10 | Tmem174 | chr2q12 |
| 1390525_a_at | -29.67 | Stra6 | chr8q24 |
| 1368718_at | -29.80 | Aldh1a7 | chr1q51 |
| 1387386_at | -29.92 | Foxj1 | --- |
| 1367804_at | -30.14 | Apcs | chr13q24 |
| 1369953_a_at | -30.62 | Cd24 | chr20q13 |
| 1392411_at | -30.73 | Tinag | chr8q24 |
| 1376292_at | -30.74 | --- | --- |
| 1387819_at | -31.09 | Cela1 | chr7q36 |
| 1382849_at | -31.48 | --- | --- |
| 1376117_at | -31.81 | Slc44a4 | chr20p12 |
| 1376953_at | -31.86 | Unc5cl | chr9q12 |
| 1384775_s_at | -32.04 | Tmprss8 | chr10q12 |
| 1385767_at | -32.33 | LOC304000 | chr11q12 |
| 1385500_at | -33.10 | RGD1561795 | chr8q11 |
| 1383983_at | -33.39 | --- | --- |
| 1391864_at | -34.23 | Enpp6 | chr16q11 |
| 1389781_at | -34.79 | Elmod1 | chr8q24 |
| 1384774_at | -34.82 | Tmprss8 | chr10q12 |
| 1391330_at | -34.93 | Asb14 | chr16p16 |
| 1395249_at | -36.19 | Snhg11 | chr3q42 |
| 1390541_at | -36.67 | Ush1c | chr1q22 |
| 1387542_at | -37.20 | Slc9a3 | chr1p11 |
| 1369491_at | -37.76 | Dao | chr12q16 |
| 1378778_a_at | -38.00 | --- | --- |
| 1368621_at | -38.09 | Aqp9 | chr8q24 |
| 1386947_at | -38.80 | Cdh1 | chr19q12 |
| 1385475_a_at | -38.85 | Stra6 | chr8q24 |
| 1368659_at | -39.43 | Agxt2 | chr2q16 |
| 1381955_at | -39.84 | Fam183b | chr10q22 |
| 1396034_at | -42.32 | Ces7 | chr19p12 |
| 1388199_at | -42.42 | Epcam | chr6q12 |
| 1393436_at | -42.49 | Scgb1c1 | chr1q41 |
| 1387223_at | -42.55 | Aadat | chr16p12 |
| 1371043_a_at | -42.73 | Pou3f3 | chr9q22 |
| 1393489_at | -43.18 | Rsb66 | chr3p12 |
| 1382127_at | -43.40 | --- | --- |
| 1368178_at | -43.87 | Pdzk1 | chr2q34 |
| 1368077_at | -44.49 | Fbp1 | chr17p14 |
| 1372190_at | -45.07 | Aqp4 | chr18p13 |
| 1381960_at | -49.00 | Ropn1l | chr2q22 |
| 1391194_at | -51.57 | Sall1 | chr19p11 |
| 1393953_at | -51.99 | Eps8l3 | chr2q34 |
| 1370592_at | -53.31 | Keg1 | chr1q43 |
| 1385679_at | -54.22 | --- | --- |
| 1383003_at | -55.67 | Lect2 | chr17p14 |
| 1391922_at | -55.88 | Helt | chr16q11 |
| 1391162_at | -57.50 | Dcdc2 | chr17p11 |
| 1381573_at | -58.82 | RGD1562107 | chr9q13 |
| 1375992_at | -59.50 | RGD1564114 | chr3p12 |
| 1369660_at | -60.30 | Defb1 | chr16p |
| 1372492_at | -60.58 | Cldn10 | chr15q24 |
| 1386671_at | -66.09 | Lrrc19 | chr5q33 |
| 1392969_at | -67.45 | --- | --- |
| 1382840_at | -70.13 | Lrrc19 | chr5q33 |
| 1382964_at | -74.32 | --- | --- |
| 1367851_at | -75.77 | Ptgds | chr3p13 |
| 1370943_at | -79.69 | Sult1c2 | chr9q11 |
| 1387139_at | -80.56 | Hao2 | chr2q34 |
| 1369317_at | -80.85 | Lcn5 | chr3p13 |
| 1370149_at | -87.90 | Asgr1 | chr10q24 |
| 1370299_at | -93.50 | Aldob | chr5q22 |
| 1384628_at | -106.57 | Iyd | chr1p11 |
| 1382669_at | -489.57 | Bin2a | chr8q13 |

**Table S2. Significantly changed genes in whole adipose tissue**

| **Probe Set ID** | **Fold change** | **Gene Symbol** | **Chromosomal Location** |
| --- | --- | --- | --- |
| 1371033_at | 6.68 | RT1-Bb | chr20p12 |
| 1385647_at | 6.11 | --- | --- |
| 1379496_at | 5.01 | --- | --- |
| 1371171_at | 4.79 | RT1-EC2 | chr20p12 |
| 1383028_at | 3.73 | Cdv3 | chr8q32 |
| 1388183_at | 3.58 | Csn1s1 | chr14p21 |
| 1370463_x_at | 3.20 | RT1-CE16 | --- |
| 1371015_at | 2.45 | Mx1 | chr11q12 |
| 1368501_s_at | 2.42 | Mcpt10 | chr15p12 |
| 1383449_at | 2.34 | --- | --- |
| 1371164_at | 2.34 | Mcpt10 | chr15p12 |
| 1370405_at | 2.18 | Mcpt1 | chr15p13 |
| 1380129_at | 2.07 | --- | --- |
| 1389408_at | 2.02 | Rrm2 | chr6q16 |
| 1371151_at | 2.01 | Cpa3 | chr2q24 |
| 1387969_at | 1.99 | Cxcl10 | chr14p22 |
| 1368706_at | 1.98 | Tm4sf4 | chr2q31 |
| 1387902_a_at | 1.97 | LOC683399 | --- |
| 1384580_at | 1.97 | C6 | chr2q16 |
| 1368762_at | 1.92 | Ubd | chr20p12 |
| 1369657_at | 1.92 | Cpa1 | chr4q22 |
| 1370382_at | 1.92 | RT1-Db1 | chr20p12 |
| 1382936_at | 1.91 | --- | --- |
| 1382314_at | 1.89 | Isg15 | chr5q36 |
| 1387283_at | 1.81 | Mx2 | chr11q21 |
| 1395615_at | 1.80 | --- | --- |
| 1385099_at | 1.77 | Scel | chr15q22 |
| 1368348_at | 1.75 | Slc6a4 | chr10q26 |
| 1371267_at | 1.73 | RT1-A1 | chr20p12 |
| 1370913_at | 1.72 | Rsad2 | chr6q16 |
| 1375501_at | 1.71 | RGD1308106 | chr1q43 |
| 1387134_at | 1.68 | Slfn3 | chr10q26 |
| 1387713_a_at | 1.68 | Fcer1a | chr13q24 |
| 1381556_at | 1.67 | Ddx60 | chr16p12 |
| 1370972_x_at | 1.65 | RT1-CE5 | chr20p12 |
| 1390236_at | 1.65 | LOC688582 | chr3p12 |
| 1388212_a_at | 1.63 | RT1-S3 | chr20p12 |
| 1367998_at | 1.62 | Slpi | chr3q42 |
| 1383032_at | 1.62 | --- | --- |
| 1382902_at | 1.62 | Herc6 | chr4q24 |
| 1379437_at | 1.61 | --- | --- |
| 1379791_at | 1.61 | Cd3e | chr8q22 |
| 1368332_at | 1.60 | Gbp2 | chr2q44 |
| 1373773_at | 1.60 | Gpm6a | chr16p11 |
| 1377379_at | 1.60 | Irf6 | chr13q27 |
| 1385799_at | 1.58 | Nxph1 | chr4q21 |
| 1373992_at | 1.57 | MGC108823 | chr18q12.1 |
| 1369668_x_at | 1.57 | Vps52 | chr20p12 |
| 1368610_at | 1.56 | Mca32 | chr10q32.1 |
| 1385969_at | 1.56 | --- | --- |
| 1388213_a_at | 1.56 | RT1-S3 | chr20p12 |
| 1386936_at | 1.56 | Grifin | chr12q11 |
| 1368780_at | 1.55 | Adrb3 | chr16q12.3 |
| 1377626_at | 1.55 | LOC690768 | chr1q43 |
| 1392776_at | 1.55 | --- | --- |
| 1385051_at | 1.55 | Gbp4 | chr2q44 |
| 1393252_at | 1.55 | --- | --- |
| 1367849_at | 1.54 | Sdc1 | chr6q14 |
| 1382546_at | 1.54 | Phf11 | chr15p12 |
| 1374726_at | 1.54 | Fndc1 | chr1q11 |
| 1387173_at | 1.54 | Cma1 | chr15p13 |
| 1374273_at | 1.53 | --- | --- |
| 1387946_at | 1.53 | Lgals3bp | chr10q32.3 |
| 1373130_at | 1.51 | Myom2 | chr16q12.5 |
| 1388071_x_at | 1.50 | RT1-EC2 | chr20p12 |
| 1368321_at | -1.50 | Egr1 | chr18q |
| 1383052_a_at | -1.51 | Zfp91 | chr1q43 |
| 1393705_at | -1.51 | --- | --- |
| 1389300_at | -1.52 | Lyrm1 | chr1q35 |
| 1387177_at | -1.52 | Vipr2 | chr6q33 |
| 1368266_at | -1.53 | Arg1 | chr1p12 |
| 1368162_at | -1.54 | Cst6 | chr1q43 |
| 1377845_at | -1.54 | Aff3 | chr9q21 |
| 1394477_at | -1.55 | LOC682058 | --- |
| 1369484_at | -1.56 | Wisp2 | chr3q42 |
| 1393795_at | -1.56 | Zeb2 | chr3q12 |
| 1368751_at | -1.56 | Kcns3 | chr6q14 |
| 1393060_at | -1.56 | Adamtsl2 | chr3p12 |
| 1370522_at | -1.57 | Gcgr | chr10q32.3 |
| 1378809_at | -1.58 | --- | --- |
| 1377334_at | -1.58 | RT1-Ba | chr20p12 |
| 1370568_at | -1.59 | Adra2c | chr14q21 |
| 1368293_at | -1.62 | Cpz | chr14q21 |
| 1383185_at | -1.63 | --- | --- |
| 1391292_at | -1.63 | --- | --- |
| 1369342_at | -1.64 | Atp7a | chrXq31 |
| 1371065_at | -1.65 | RT1-Bb | chr20p12 |
| 1388456_at | -1.65 | S100a1 | chr2q34 |
| 1376174_at | -1.65 | Serpina11 | chr6q32 |
| 1372226_at | -1.67 | --- | --- |
| 1380209_at | -1.68 | --- | --- |
| 1368373_at | -1.71 | Rgs7 | chr13q24-q25 |
| 1392166_at | -1.72 | --- | --- |
| 1385682_at | -1.74 | Vit | chr6q11 |
| 1387073_at | -1.78 | Snap25 | chr3q36 |
| 1378889_at | -1.78 | --- | --- |
| 1383557_at | -1.79 | --- | --- |
| 1370912_at | -1.79 | Hspa1a | chr20p12 |
| 1387039_at | -1.80 | Gpc1 | chr9q36 |
| 1367847_at | -1.80 | Nupr1 | chr1q36 |
| 1388357_at | -1.83 | RGD1566401 | chr6q32 |
| 1392159_at | -1.85 | --- | --- |
| 1374622_at | -1.87 | --- | --- |
| 1384743_at | -1.89 | --- | --- |
| 1368281_at | -1.90 | Dpep1 | chr19q12 |
| 1395335_at | -1.92 | --- | --- |
| 1377008_at | -2.02 | RGD1566401 | chr6q32 |
| 1375043_at | -2.02 | Fos | chr6q31 |
| 1379368_at | -2.03 | Bcl6 | chr11q23 |
| 1368282_at | -2.05 | Dpep1 | chr19q12 |
| 1378015_at | -2.07 | Ccl21b | chr5q22 |
| 1382882_x_at | -2.13 | --- | --- |
| 1370902_at | -2.18 | Akr1b8 | chr4q22 |
| 1367581_a_at | -2.36 | Spp1 | chr14p22 |
| 1396714_at | -2.39 | --- | --- |
| 1377429_at | -2.40 | Lpo | chr10q26 |
| 1376711_at | -2.45 | Cldn11 | chr2q24 |
| 1368021_at | -2.62 | Adh1 | chr2q44 |
| 1387631_at | -2.66 | Hpgd | chr16p11 |
| 1379065_at | -2.67 | Serpina12 | chr6q32 |
| 1387022_at | -2.72 | Aldh1a1 | chr1q51 |
| 1377133_at | -2.74 | LOC680687 | chr2q43 |
| 1387273_at | -2.76 | Il1rl1 | chr9q21 |
| 1376191_at | -2.81 | Hpgd | chr16p11 |
| 1384013_at | -3.64 | --- | --- |
| 1388203_x_at | -4.98 | RT1-A3 | chr20p12 |
| 1367556_s_at | -5.10 | Alb | chr14p22 |
| 1367555_at | -5.17 | Alb | chr14p22 |
| 1388202_at | -5.42 | RT1-EC2 | chr20p12 |
| 1368887_at | -6.06 | --- | --- |
| 1369202_at | -6.35 | Mx2 | chr11q21 |
| 1368733_at | -6.47 | Sult1e1 | chr14p21.3-p21.2 |
| 1367923_at | -7.54 | Acsbg1 | chr8q22-q24 |
| 1381593_x_at | -22.53 | RT1-Ba | chr20p12 |
| 1368578_at | -25.43 | Hsd3b1 | chr2q34 |
| 1368569_at | -35.86 | Akr1b7 | chr4q22 |
